# Rising snowline altitudes across Southern Hemisphere glaciers from 2000 to 2023

**DOI:** 10.1038/s41598-025-19486-6

**Published:** 2025-10-13

**Authors:** Mia MacFee, Jonathan L. Carrivick, Duncan J. Quincey

**Affiliations:** School of Geography and water@leeds, University of Leeds, Woodhouse Lane, Leeds, LS2 9JT West Yorkshire England

**Keywords:** Cryospheric science, Climate-change impacts

## Abstract

Understanding of Southern Hemisphere glacier response to climatic changes is limited by a paucity of direct observations. However, glacier Equilibrium Line Altitudes (ELAs), which are determined by air temperature and precipitation and represent glacier mass balance, can be approximated using remote sensing of end of summer snowline altitudes (SLA_EOS_). This study shows that SLA_EOS_ increased on the majority of 6364 glaciers and by up to 7.18 m yr^−1^ between 2000 and 2023 across five Southern Hemisphere mountain regions. Over this period anomalies from the multi-decadal mean SLA_EOS_ became predominantly positive for all regions. In the most extreme cases, the rate of SLA_EOS_ rise accelerated by up to x 4.0 in the western Southern Alps of New Zealand when comparing pre- and post-2010 rates of change. Contrastingly, the western Antarctic Peninsula and the western Southern Andes experienced declining SLA_EOS_ and slowing rates of change at -1.0 and − 8.1 m yr^−1^, respectively. These spatio-temporal patterns reveal the interplay of temperature and precipitation, and the effects of them on a wide variety of mountain glaciers and ice cap outlet glaciers, with implications for understanding glacier response times, committed ice losses and overall meltwater production in the coming decades.

**Supplementary Information:**

The online version contains supplementary material available at 10.1038/s41598-025-19486-6.

## Introduction

**Fig. 1:**
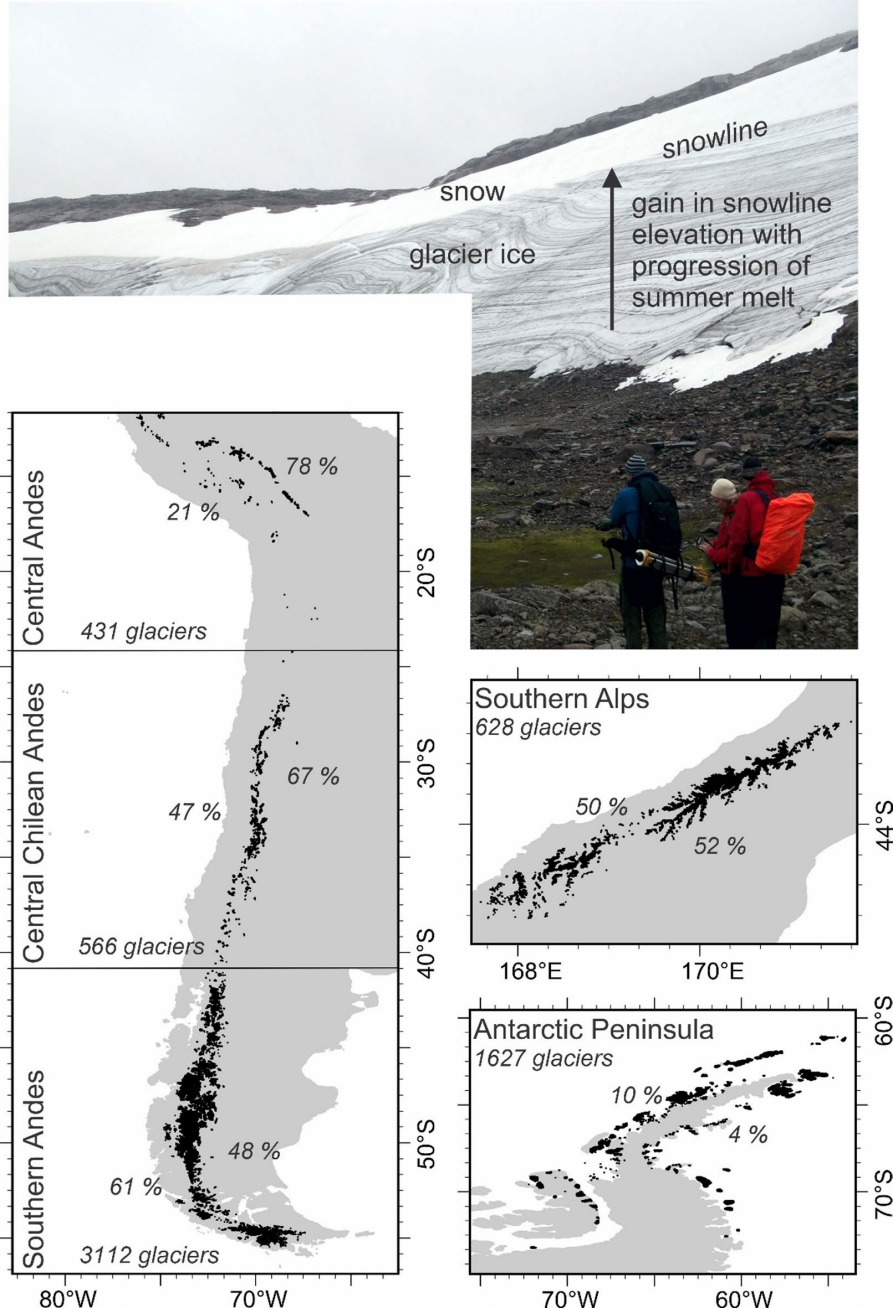
Annotated field photo showing end of summer snow line (SLA_EOS_) and multi-panels depicting the spatial coverage of our analysis; glaciers in black. The number of glaciers in each region depended on filtering glacier sizes and filtering satellite images for timing and cloud cover. Annual counts of glaciers included per sub-region are given in Figure SI_8 and the % area coverage per sub-region is annotated on this figure. The maps in this figure were made using ArcGIS Pro software https://www.esri.com/en-us/arcgis/products/arcgis-pro/overview.

Just 10% of glaciers with direct mass balance monitoring are located within the Southern Hemisphere^1^. Understanding of Southern Hemisphere glacier response to climatic changes is therefore lacking spatio-temporal coverage, and more specifically, inter-annual and seasonal changes of glacier mass balance remain poorly quantified^2^. Remote sensing geodetic mass balance methods have revealed widespread glacier mass loss^3^ but have also highlighted considerable spatial and temporal variability in patterns and rates. An alternative solution for evaluating glacier response is to use annual end-of-summer snowline altitudes (SLA_EOS_) as a proxy for the glacier Equilibrium Line Altitude (ELA) and thus long-term glacier mass balance^4–7^. SLA_EOS_ can be defined as the lowest elevation on a glacier where snow cover remains at the end of a summer season (Fig. 1). Annual SLA_EOS_ depends directly on air temperature and precipitation and more specifically on the surface energy balance, which is composed of turbulent heat fluxes; sensible heat due to temperature changes, latent heat due to phase changes e.g. solid to liquid for melting, and radiation fluxes; dominated by shortwave incoming radiation. SLA_EOS_ can either be retrieved from glacier mass balance monitoring, or automatically from satellite images. However, there are only seventy glaciers in the world with long-term ELA records^8^ and just seven of them are located in the Southern Hemisphere. There is therefore a research gap for developing automatic retrieval of annual snowline altitudes from satellite images to determine the ELA of Southern Hemisphere glaciers. The aim of this study is to determine spatial patterns and temporal trends of SLA_EOS_ for thousands of glaciers across the Southern Hemisphere from 2000 to 2023.

### Study area

Our study encompasses mountain regions across the Southern Hemisphere, namely; Southern Alps of New Zealand, Antarctic Peninsula, Central Andes, Central Chilean Andes and the Southern Andes. These regions are split between east and west to recognise major morpho-climatic zones (Fig. SI_1).

### Methodology to gain spatio-temporally resolved end of summer snowline altitudes (SLA_EOS_)

This study developed a novel approach using Google Earth Engine (GEE) to retrieve, filter and analyse satellite images to firstly classify snow cover versus bare ice, and to then derive the elevation of the boundary between those parts for multiple glaciers for multiple years. Our approach enables coverage of thousands of glaciers across multiple world regions and spanning several decades. Specifically, the workflow of Li et al.^11^ was expanded to gain SLA_EOS_ values across a sequence of years for multiple glaciers simultaneously. Our workflow was entirely within Google Earth Engine and includes image and elevation data processing, snow classification and SLA_EOS_ identification, was in GEE (Fig. SI_2), and iterated across a list of years and a list of glaciers. Our datasets are described in our Table SI_1).

Near-annual SLA_EOS_ values were retrieved for 6364 glaciers across the Southern Hemisphere between 1 st January 2000 and 31 st December 2023 and at 30 m horizontal resolution (Fig. 1, Table SI_2). Our results are presented in a conservative manner, only including glaciers with ten or more observations of annual SLA_EOS_ and combining glaciers in both spatially-aggregated and temporally-averaged forms, thereby mitigating locally-specific glaciological or topographic controls, uncertainty in the end-of-summer timing, and inter-annual variability, for example. Overall, major spatial patterns and temporal trends are discerned in Southern Hemisphere SLA_EOS_ that are associated with climate change. Inter- and intra-region spatial variability and temporal trends are examined for the full study period 2000 to 2023, as well as for sub-periods 2000 to 2010 and 2011 to 2023.

### Image and elevation data processing

Landsat collections were filtered to the period 2000 to 2023 and to the end-of-summer timeframe for each region. For the Andes and Southern Alps, previous studies support the timeframe of mid-February to early-April^12^. For the Antarctic Peninsula, annual peak snow melt has been documented in January^13^. Therefore, SLA_EOS_ values reported in this study are the maximum of all SLAs retrieved from all available images between 01/02 and 30/04 for the Andes and Southern Alps, and between 01/01 and 30/04 for the Antarctic Peninsula, to allow for fluctuations in the timing of maximum SLA between years. East African and Indonesian glaciers are too small and too few to yield statistically robust spatial patterns or temporal trends and so are omitted from our study.

Filtering Landsat images were filtered to retain only those images with < 50% cloud cover. Images were then clipped to each glacier outline and the C Function of Mask (CFMask) algorithm was applied to mask any cloud-covered pixels that could have a similar reflectance to snow. Erroneous pixels (usually with a value > 65535) were also masked. The valid pixel ratio of every image for a given glacier was calculated, and only images with > 65% visible pixels remaining following cloud masking were retained for further analysis^11^. This step also minimised the effect of Landsat 7 scan line corrector error that created tracks of invalid image pixels over some glaciers.

The AW3D30 v. 3.1 Digital Elevation Model (DEM), which is a mosaic dataset composed of data from 2006 to 2011 and with a median date of 2009, was static (unchanged) throughout our study and workflow, enabling direct comparison of SLAs obtained for each year per glacier. Functions were run to calculate the minimum and maximum elevation of individual glaciers, and to generate a List sequence of contour elevations between these values at 20 m intervals.

### Snow classification

Images in our filtered collection were individually processed to detect snow-covered parts of glaciers. Each image was topographically corrected by sun-canopy-sensor correction, SCS + C^15^ using GEE-based code adapted from top-of-atmosphere topographic correction methods^15^ and surface reflectance topographic correction methods^16^. Topographic correction reduces underestimation of snow-covered pixels caused by topographic shadow^17^ and the SCS + C method is well-supported by previous studies, which encourage its use for mountainous environments^18,19^. Although part of image preparation, topographic correction took place at this stage of our workflow for efficiency (versus correcting entire image collections simultaneously).

As the use of different band ratios, NDSI and NIRSWIR, currently divides automated snowline analyses^20,21^ annual mean SLA_EOS_ was initially calculated in this study using both NDSI and NIRSWIR ratios and compared them to those collected by the New Zealand aerial survey^10^ (Fig. SI_10). SLA_EOS_ results from the NIRSWIR approach showed a weak positive correlation with aerial survey results (*r* = 0.19), while results from the NDSI approach showed moderate negative correlation (*r* = −0.44). Although aerial survey data cannot necessarily be treated as ground-truth due to the uncertainty introduced with converting the snowline viewed on an oblique image to an elevation, this study chose to use the NIRSWIR Otsu method, interpreting the problems the NIRSWIR for each glacier image by using bands 4 and 5 in Landsat 4 TM/5 TM/7 ETM+, and bands 5 and 6 in Landsat 8 OLI.

The Otsu method^21^ of binary image segmentation was applied to classify the image into ‘snow’ and ‘ice’ pixels using a histogram from the NIRSWIR. This statistical algorithm identifies glacier-specific classification thresholds, by identifying the maximum between-class variance, corresponding to the maximum between-sum-of-squares of pixel values in each of our filtered images (Fig. SI_3). Otsu has been applied in several automated snowline detection studies e.g^23^. as it is favoured over manual and semi-automated methods for adaptively identifying snow-ice boundaries over large numbers of clipped images^6,7,23^. Clipping images to glacier outlines was necessary because this method assumes each image contains only snow and ice pixels so if other light-absorbing land cover types (e.g., rock or vegetation) are erroneously contained within the glacier area, they will likely be classified as ‘ice’ and cause a shift in the Otsu threshold value.

### SLA_EOS_ identification

Topographic and climatic factors can cause hollows, shadows, avalanche deposits and wind-blown snow to create perforated snow cover in the vicinity of a glacier snowline, so that the snowline may be non-continuous and span a range of altitudes^6^. Consequently, the World Meteorological Organisation recommends that SLA_EOS_ be represented as an altitudinal zone rather than a specific altitude. The classified glacier image was then separated into 20 m elevation bins using the list of contours created in data preparation, and the snow cover ratio (SCR) of each bin was calculated from its ‘snow’ and ‘ice’ pixel counts.

A function was then applied to identify the lowermost set of 5 consecutive elevation bins which each had SCR > 0.5 (snow coverage > 50%). If a set was identified, SLA_EOS_ was taken as the average elevation of the lowest bin in that set. If unidentified, the required number of bins in a set was reduced by 1, until the condition for SCR was met. If no elevation bin with SCR > 0.5 was identified, SLA_EOS_ was returned as a null value. When there were multiple usable images for a glacier in a given year, multiple SLA_EOS_ values were produced. In this case, the maximum SLA_EOS_ value was taken. When only one SLA_EOS_ value was produced it was not recorded, meaning that every SLA_EOS_ result used in this study is a maximum SLA_EOS_ compared to at least one other SLA_EOS_ result for that glacier and that year. Temporal trends were identified with a best-fit linear regression through the annual regional and sub-regional SLA_EOS_ values. The significance of that trend was assessed with a t-test on the slope coefficient (whether it being different to zero).

### Accuracy and uncertainty

Our remotely-sensed observations are evaluated in three independent ways. Firstly, our SLA_EOS_ are compared to the aerial survey dataset of annual SLA_EOS_ of ~ 50 glaciers across the Southern Alps^5,9,10^ (SI).

Secondly, our SLA_EOS_ are compared to ELAs calculated from direct mass balance measurements on the 6 reference glaciers in the Southern Hemisphere (Table SI_3). The latter a yielded positive correlation (*r*^*2*^ = 0.76) between our sub-regional median rate of SLA_EOS_ change and change in ELA. Martial Este Glacier in the Southern Andes is not well-represented by the regression line, and as it is a strongly maritime glacier it could be considered as an anomaly and if it is removed then the correlation increases to *r*^*2*^ = 0.95 (Fig. SI_12).

Thirdly, random point assessments were made for each sub-region, selecting glaciers with a range of aspects and assessing uncertainty for two separate years. Both Landsat 7 ETM + and Landsat 8 OLI were represented. Each assessment comprised 100 points (per clipped image; Fig. SI_4) classified visually and manually into snow, ice, and N/A (blank image) groups, by interpretation of false-colour images (bands 5, 4, and 3 for Landsat 5TM/7ETM+, and bands 6, 5, and 4 for Landsat 8 OLI), and consideration of the likely locations of accumulation and ablation zones (Fig. SI_4). These interpretations were then compared to our automatic SLA_EOS_ classifications to calculate percentage uncertainty. The mean accuracy of snow classification was 87.4 ± 6.2% across all regions and all years assessed. Landsat 7 ETM + images increased the number of N/A validation points due to gaps associated with scan Line error, but uncertainty did not vary between cases where Landsat 7 ETM + images were present (87.5%) and cases where only Landsat 8 OLI images were present (87.2%). Overall, our estimated mean accuracy of snow classification was 87.4% across all regions and all years. An uncertainty was assigned to each median SLA_EOS_ (per year, per sub-region) of one standard deviation.

Uncertainty varied more strongly between regions and particularly across the four Andean regions, with the Central Chilean region displaying highest (93.9%) snow classification accuracy (Fig. SI_5). This outcome could be expected due to regional differences in cloud cover^27^ (Fig. SI_6), which made for increased image clarity in the Central Chilean region. We noted the problems introduced by bush fire ash and topographic shading on our classifications, for example (Fig. SI_7).

Sources of uncertainty within our automated SLA_EOS_ calculation arise from (i) vertical accuracy of elevation data, (ii) accuracy of satellite images used in glacier outline generation and snowline analysis, and (iii) accuracy of the SLA_EOS_ identification method. Assuming that the uncertainty sources are uncorrelated, SLA_EOS_ uncertainty was calculated as the Root Mean Square Error (RMSE) of the sum of the uncertainty in the (i) ALOS AW3D30 v. 4.1 DEM, which is ± 5 m vertically, (ii) spatial referencing of Landsat satellite images, considered as ½ pixel size ± 15 m^28^and (iii) the zonal method used for SLA_EOS_ identification, where SLA_EOS_ is taken as the average elevation of a 20 m bin. Overall, for any individual SLA_EOS_ absolute uncertainty is ± 18.7 m. For our groups of glaciers (space and time; Figs. SI_8, SI_9) typically the standard error is > 15 m in any year for any sub-region and approximately one tenth of that when aggregating into 5-year bins.

### Results: trends in SLA_EOS_ between year 2000 and 2023

**Fig. 2:**
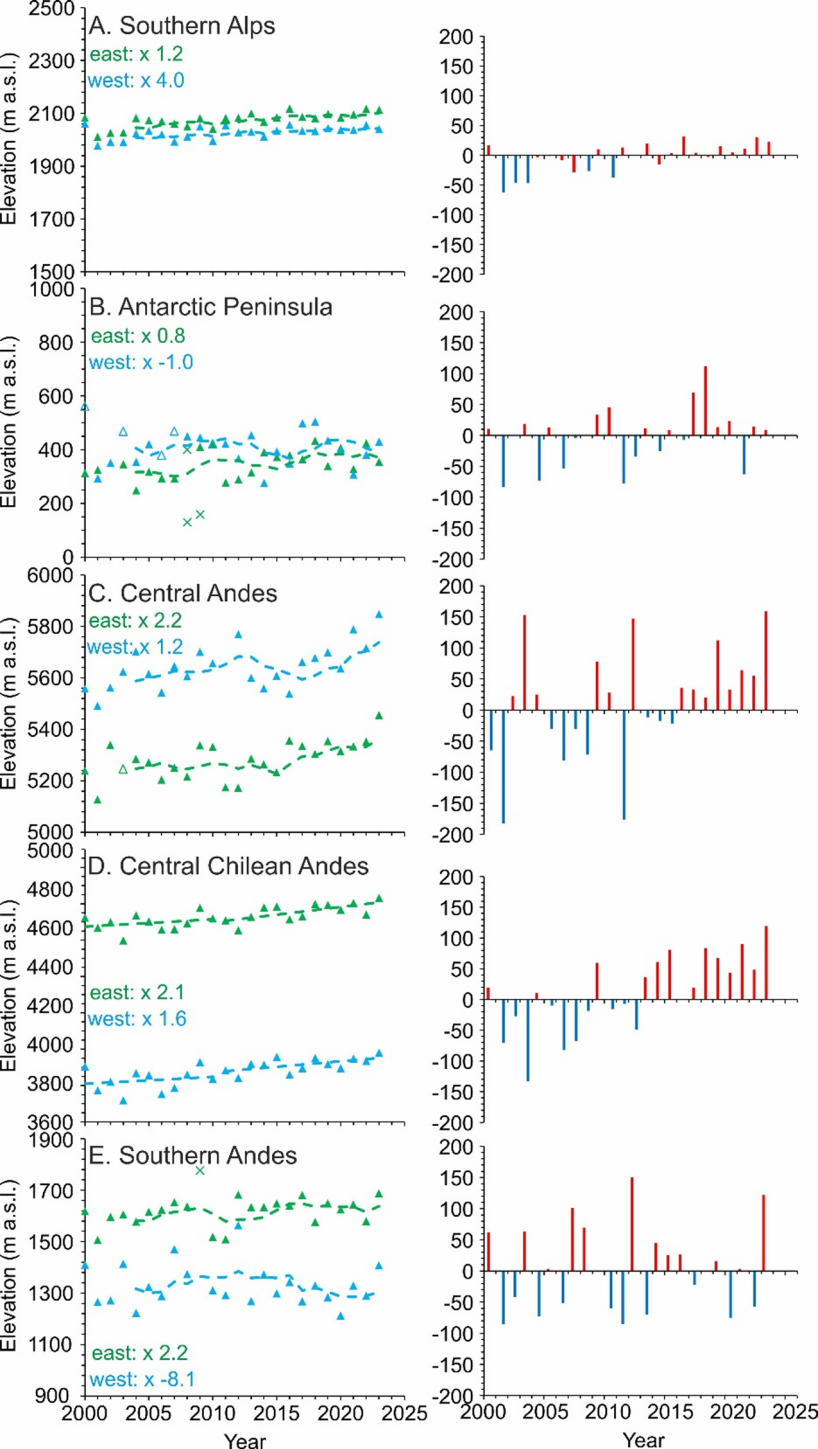
Time series of SLA_EOS_ for each major glaciated region of the Southern Hemisphere, split east and west (left column), and annual anomalies; differences with the study period mean (right column). The quantity in bold inset to the SLA_EOS_ plots denotes the acceleration when comparing pre-2010 to post-2010 rates of change. Note all y scales for SLA_EOS_ span 1000 m except that for the Central Chilean Andes. Open triangles are where *n* < 10. Dashed Lines are a 5-year moving average. Region-wide annual anomalies are calculated relative to the 2000 to 2023 mean.

**Fig. 3:**
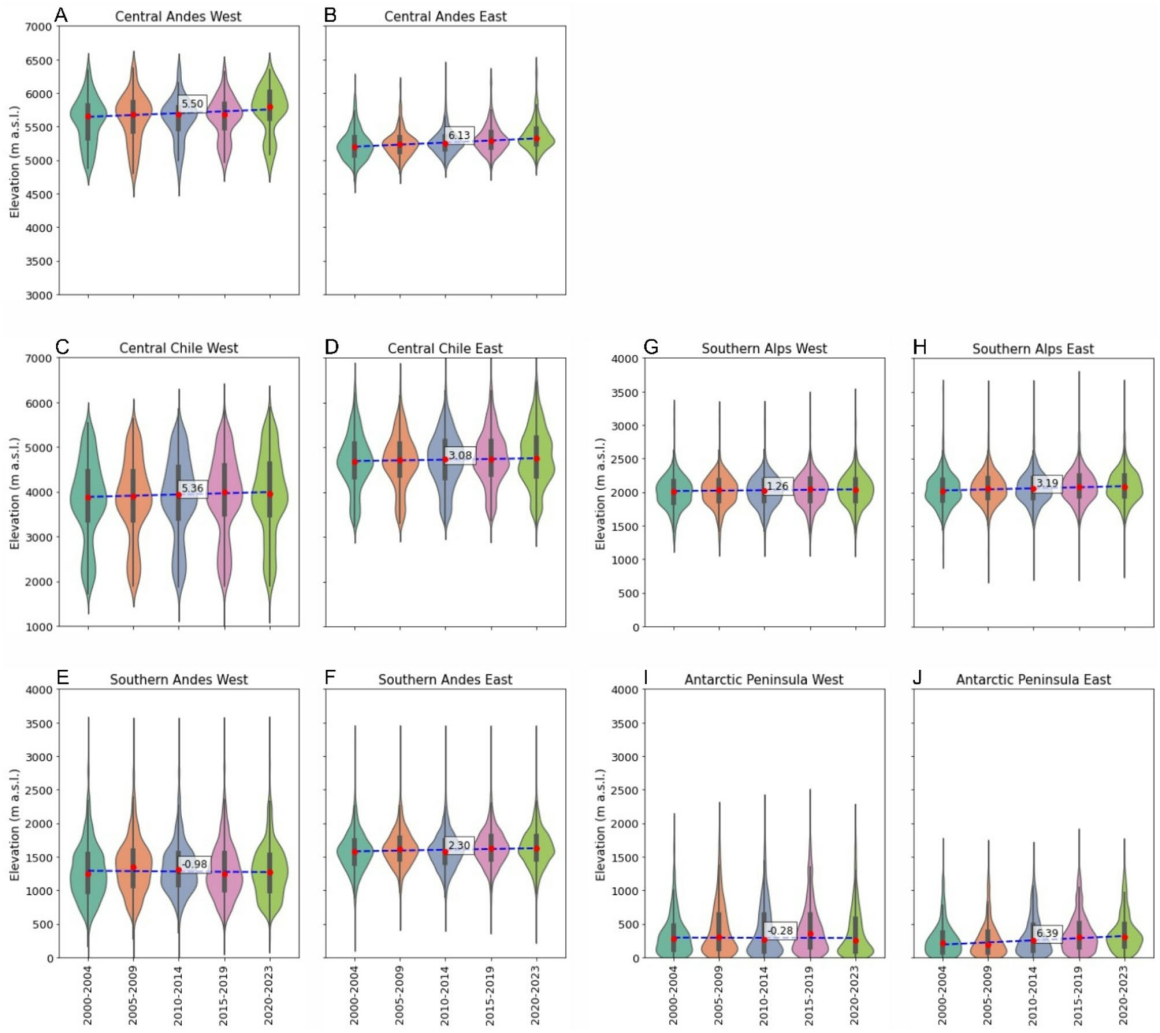
Temporal distribution of our SLA_EOS_ within 5-year bins by sub-region, with median and interquartile range represented by the horizontal bar and box, respectively, a linear trend through the medians indicated by the dashed line and the gradient of that annotated (m yr^−1^).

Average SLA_EOS_ increased across the Southern Hemisphere between 2000 and 2023 (*p* = 0.01), especially between 2011 and 2023 (Figs. 2 and 3). Only the Southern Andes and the Antarctic Peninsula experienced region-wide overall decline in median SLA_EOS_ of −0.98 m yr^−1^ and − 0.28 m yr^−1^, respectively (Fig. 3I and J), caused by negative values in the western sub-regions only (Fig. 2B and E).

The Southern Alps of New Zealand had the greatest west – east similarity in absolute SLA_EOSs_ (< 100 m) of any region (Fig. 3G and H), the most consistent pattern between east and west, and the lowest inter-annual variability (Fig. 2A). Between 2000 and 2023, SLA_EOS_ rose in the western and eastern parts of the Southern Alps by 1.26 ± 0.21 m yr^−1^ (*p* = 0.049) and 3.19 ± 0.20 m yr^−1^ (*p* = 0.002), respectively (Fig. 3) SLA_EOS_ anomalies, relative to the study period mean, have been consistently slightly positive across the Southern Alps for the last 13 years since 2010 (Fig. 2A).

The Antarctic Peninsula had SLA_EOS_ rates of change of −0.28 ± 0.32 m yr^−1^ in the west (insignificant *p* = 0.921), and contrastingly the east showed the fastest rate of rise of any region at 6.39 ± 0.39 m yr^−1^ (*p* = 0.013) (Fig. 3). There was a slight deceleration in the rate of SLA_EOS_ rise in the west (x −1.0) and only a slight increase in the east (x 0.8) of the Antarctic Peninsula comparing the decade 2000 to 2010 with the decade 2011 to 2023 (Fig. 2B).

Spatio-temporal patterns in SLA_EOS_ varied in magnitude considerably across the Andes. Aside from the aforementioned west Southern Andes, the other Andes regions all showed accelerated rates of SLA_EOS_ rise, when comparing rates between 2000 and 2010 with those of 2010 to 2023. Region-wide median rates in the west and the east of the Central Andes were not statistically significant at 5.5 ± 2.07 m yr^−1^ (*p* = 0.120) and 6.13 ± 0.25 m yr^−1^ (*p* = 0.052), respectively (Fig. 3A and B). The Central Chilean Andes had the greatest west – east differences in absolute SLA_EOS_ of any region, typically of c. 900 m, and also the greatest inter-region variability (largest inter-quartile range; Fig. 3C and D). The west and east region-wide median rates were 5.36 ± 0.95 m yr^−1^ (*p* = 0.003) and 3.08 ± 0.38 m yr^−1^ (*p* = 0.002), respectively (Fig. 3C and D). In the Southern Andes the west and east region-wide median rates changed insignificantly by −0.98 ± 0.13 m yr^−1^ (*p* = 0.486) and 2.30 ± 0.07 m yr^−1^ (*p* = 0.119), respectively, between 2000 and 2023 (Fig. 3E and F). SLA_EOS_ anomalies in the Central Andes and Central Chilean Andes regions have been increasingly positive, compared to the study period mean since 2015 and 2012, respectively (Fig. 2C and D).

### Spatial patterns and variability

**Fig. 4:**
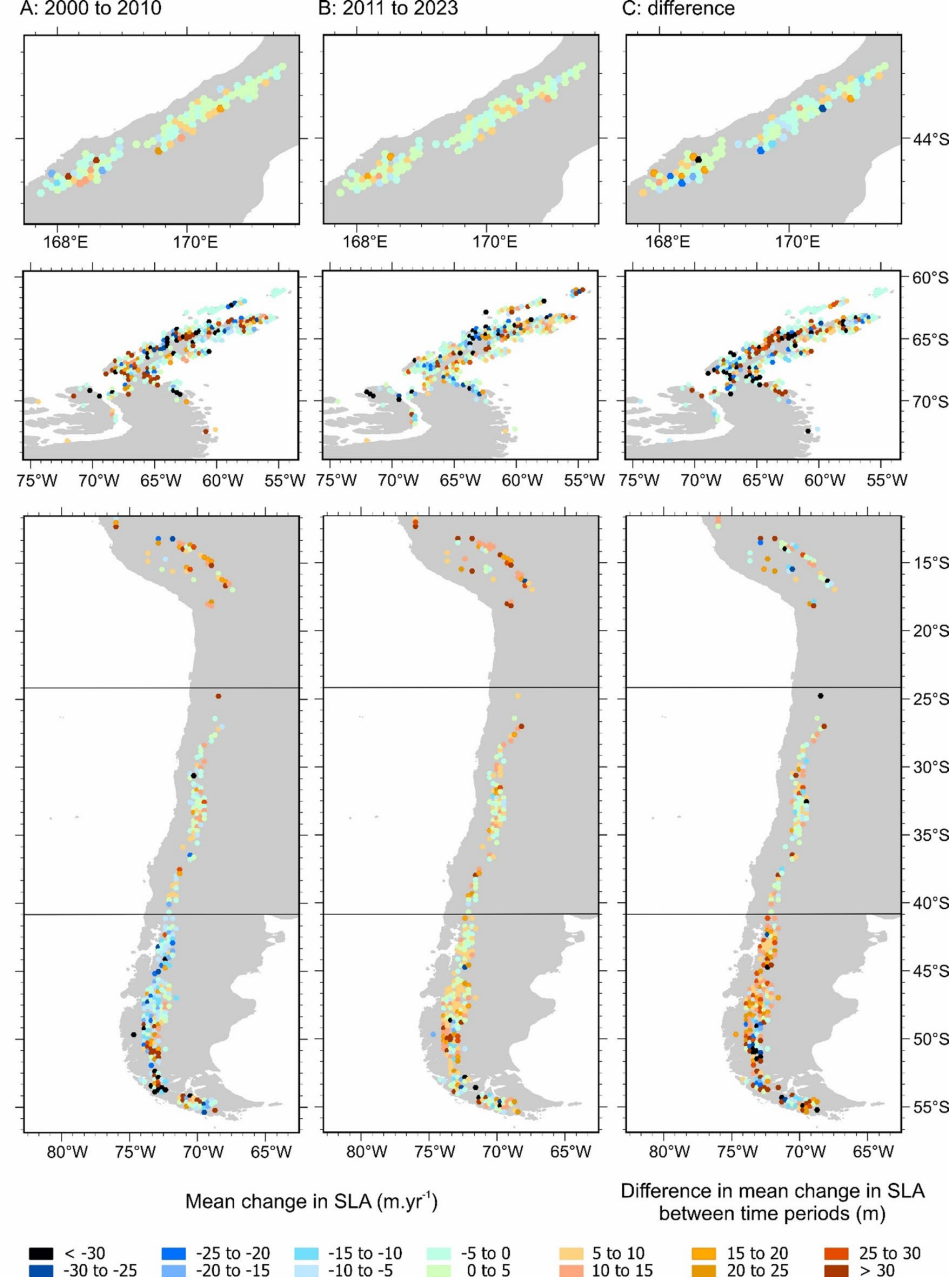
Spatial pattern of the mean rate of change in SLA_EOS_ for all glaciers within each tessellation cell from 2000 to 2010 (**A**) and from 2011 to 2023 (**B**). The difference in rate between the two time periods (**C**) uses the same colour scale. The maps in this figure were made using ArcGIS Pro software https://www.esri.com/en-us/arcgis/products/arcgis-pro/overview.

In the Southern Alps, SLA_EOS_ persistently increased in elevation from south-west to north-east. The eastern side of the Southern Alps main divide showed consistently higher mean SLA_EOS_ than the west over the study period, but typically by only ~ 75 m (Fig. 4A and B). However, there is no discernible spatial pattern in the mean rate of SLA_EOS_ change across the Southern Alps during the entire study period 2000 to 2023 nor in the difference between the two sub time periods (Fig. 4C).

SLA_EOS_ was highly variable spatially across the Antarctic Peninsula, with inland parts having higher SLA_EOS_ (> 1100 m a.s.l.) than most coastal parts (< 200 m a.s.l., including the periphery South Shetlands and South Orkneys). The north-east coast of the Peninsula showed notably higher SLA_EOS_ (~ 300 to 800 m a.s.l.) than other coasts, particularly between 2000 and 2010 (Fig. 4A). Western parts of the Peninsula had higher mean SLA_EOS_ than eastern parts except in years 2010, 2014, 2016 and 2020. SLA_EOS_ increased significantly (5.2 m yr^−1^; *p* = 0.013) in the east, whereas it decreased non-significantly (−0.3 m yr^−1^; *p* = 0.921) in the west. Comparing the rates of the two sub time-periods, the north-east of the Peninsula had rates of SLA_EOS_ that increased faster than in the south-west (Fig. 4C).

The acceleration in SLA_EOS_ rise, as defined by an increased rate of change when comparing rates between 2000 and 2010 with rates from 2010 to 2023, has been much more pronounced in the west (x 4.0) of the Southern Alps than the east (x 1.2) (Fig. 2A). SLA_EOS_ rise has been really remarkably consistent across the Andes east sub-regions, whilst varying greatly in the west Andes sub-regions (Fig. 4a and B). The Central Andes, Central Chilean Andes and Patagonia had mean rates of SLA_EOS_ change that were generally positive (Fig. 4A, B and C), whereas Tierra del Fuego had mean rates of change in SLA_EOS_ that were negative during the study period 2000 to 2023 (Fig. 4C).

## Discussion

Our comparisons for each sub-region of the multi-decadal mean SLA_EOS_ (Fig. 3), inter-annual anomalies (Fig. 2) and the difference between the pre- and post-2010 mean SLA_EOS_ (Fig. 2) all reveal rising and accelerated rates of rise. The sub-regions that are exceptions are western Antarctic Peninsula and western Southern Andes where decreases in SLA_EOS_ occurred, probably reflecting the role of effective precipitation; with rising temperatures remaining below zero and thus increasing snowfall^29^. There is therefore a pervasive pattern across most of Southern Hemisphere glaciers of diminishing glacier accumulation areas, enlarging glacier ablation areas and hence increasingly negative glacier mass balance, which has implications for committed meltwater production in the coming decades. We tested for statistically significant correlations of SLA_EOS_ with summer (December to March) air temperature and snowfall but the trends in the climate data are slight (Fig. SI_13, Table SI_4) reflecting high variability even within sub-regions. Such high inter-regional variability could reflect the importance of micro-climate; e.g. warming crossing the zero degree threshold for solid versus liquid precipitation, and control of local topographical and glaciological factors (e.g. Fig. SI_7).

Where glaciers are ice cap type, such as the glacierised volcanoes and Patagonian ice fields of South America, many parts of the mainland Antarctic Peninsula and surrounding islands, rising SLA_EOS_ intersecting plateau edges will cross a tipping point whereby large proportions of the glacier accumulation area will be exposed to melt, fundamentally changing glacier geometry and velocity regimes^30,31^as well as lowering glacier albedo and raising the possibility of extremely rapid mass loss^32,33^.

**Fig. 5:**
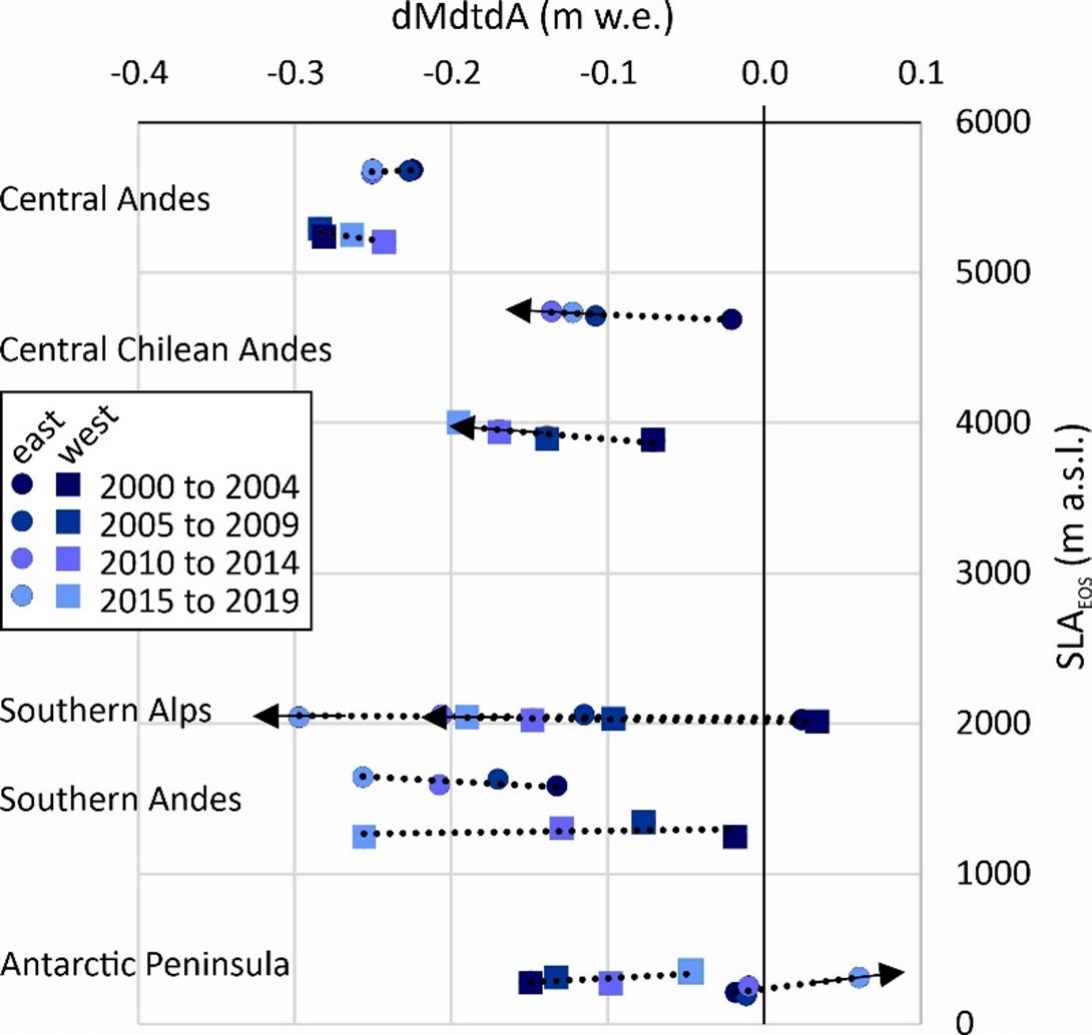
Comparison of glacier mass balance (dMdtdA)^3^ with our SLA_EOS_ for 5-year bins and for each sub-region. Where our SLA_EOS_ trends are significant, the dotted trend Line has an arrow centred on the 2015 to 2019 data point.

The positive correlations of these SLA_EOS_ rises with geodetic mass balance^3^ (Fig. 5) highlights that inter-regional differences in SLA_EOS_ across the Southern Hemisphere span > 5500 m in elevation, and that is well-known to reflect extremely diverse climate - topography interactions and hence glacier types. In particular, the intra-regional differences in SLA_EOS_ is greatest in the Central Chilean Andes (Fig. 3), but through time, in 5-year bins, the greatest mass balance range occurs in the Southern Alps (Fig. 5). More generally, Fig. 5 suggests that the response of glaciers in the Southern Hemisphere to changes in SLA is heterogeneous. Glaciers situated at very high-elevation (Central Andes) have experienced some of the fastest changes in SLA_EOS_, but the impact on mass balance has been low. Glaciers in the mid- and high-elevation range (Central Chilean Andes, Southern Andes and the Southern Alps) have experienced mostly rapid rises in SLA_EOS_, and the impact on mass balance has been high. Glaciers at the lowest elevations (i.e. mostly on the Antarctic Peninsula) have seen changes in SLA_EOS_ that vary, but the mass balance changes have been moderate, as well as positive. These groups primarily reflect the dominant climate regime within each region, along with glacier-specific factors such as area, slope and elevation, which together define the mass balance gradient (rate of input vs. output) and therefore a glacier’s sensitivity to changes in accumulation and ablation^34–36^.

### Associations of SLAs with climate

In the Southern Alps the spatial pattern of change comprising increasing SLA_EOS_ from south-west to north-east is supported by analysis of the independent aerial survey dataset^5,9,10^ (Figs. SI_10, SI_11). Lower SLA_EOS_ on the west of the main divide can be explained by the synoptic climate control on snow accumulation. Specifically, the steep elevation increase from sea level at the west coast to > 3000 m a.s.l., coupled with prevailing westerly air circulation over the Tasman Sea, produce a strong east-west orographic precipitation gradient^37^ and ~ 30% greater snowfall on glaciers to the west when compared to those on the east of the topographic divide^38,39^. The north-south pattern in SLA_EOS_ is likely due to a weak but persistent latitudinal air temperature gradient^40,41^ and possibly also to the more southerly aspect of southernmost glaciers that affects solar radiation intensity^5^.

The lowering SLA_EOS_ between 2000 and 2008 and increases thereafter (Fig. 2A), coincides with a period of well-documented^42^ glacier advance between 1983 and 2008 relating to a period of reduced air and sea surface temperatures (SST). Mass balance simulations^42^ confirm that year 2000, when a positive anomaly is observed on the SLA_EOS_ (Fig. 2A), had the most negative Southern Alps mean mass balance on record from 1972 to 2011, attributed to positive temperature anomalies enhancing snow and glacier ice ablation in the 1999/2000 summer. Another peak in SLA_EOS_ in 2016 (Fig. 2A) can also be explained climatically; a strong ENSO in 2015/2016 austral summer^43–45^ caused air temperatures across New Zealand > 1.2˚C above average (NIWA, 2016); the Tasman Sea experienced an extreme and prolonged marine heatwave^10,46–48^and elevated precipitation to the West Coast and Tasman zones^49^.

The pattern of SLA_EOS_ across the Antarctic Peninsula from 2000 to 2023 is closely associated with glacier/terrain elevation. Coastal sub-regions had consistently lower SLA_EOS_ (Fig. 3B) than inland sub-regions and it is these coastal parts of the Antarctic Peninsula and surrounding islands that host low-lying marine-terminating glaciers and ice cap outlet glaciers^50^. Our finding of generally higher SLA_EOS_ in the west (Fig. 2B) contradicts claims that glacier ELAs are higher in the east of the Antarctic Peninsula^51,52^ and also suggestions of a west-to-east precipitation decline^53^ arising from an orographic influence of the inland plateau on westerly moisture transport^54^.

Our results from the Antarctic Peninsula imply that temporal trends in SLA_EOS_ accelerated from 2011 with a faster rate of rise in the east, slower rate of rise in the west, and overall more positive SLA_EOS_ anomalies from 2017 (Fig. 2B). This temporal trend suggests that the well-reported temperature sensitivity of these glaciers^55–57^ is reflected in SLA_EOS_ as the Antarctic Peninsula experienced overall cooling from the late 1990 s to the mid-2010s^58,59^followed by warming to 2020^61^. The decreased rate of SLA_EOS_ rise in the west can be explained by increased temperatures bringing more snowfall, as has been credited for especially low glacier ELA on King George Island and on the Antarctic Peninsula^61,62^.

The Central Andes show SLA_EOS_ values that are higher overall for western glaciers than for eastern glaciers, but this spatial pattern is reversed for the Central Chilean and Southern Andes (Fig. 2C, D, E and F). The SLA_EOS_ east-west pattern(s) across the Andes (Fig. 3A and B) are directly aligned with the direction of east-west precipitation gradients with latitude. North of ~ 23–29˚S, moisture transport within the Central sub-regions primarily originates in the Atlantic and circulates easterly to the Andes, which themselves act as a topographic barrier largely preventing moisture transport to western glaciers^63,64^. South of 29˚S, Pacific moisture transport becomes more prominent^64^although between 29˚S and 35˚S the east-west precipitation contrast is reduced^65^ as easterly circulation becomes more dominant in summer^66^. South of 35˚S, strong westerly circulation delivers high quantities of precipitation to western glaciers, enhanced by orographic effects, which minimise precipitation delivery to the east^63,65^.

The Central Andes SLA_EOS_ lacks a significant trend, characterised instead by very high inter-annual variability (Fig. 2D). The multiple short periods of snowline lowering (particularly 2004 to 2006, but also 2013 to 2015 and 2016 to 2018) (Fig. 2D) partly correspond with the negative SLA trend noted at Zongo Glacier, Bolivia, from 1996 to 2006^68^^69^,. SLA_EOS_ variability in the Central Andes is seemingly linked with ENSO fluctuations; mean SLA_EOS_ was very low in 2001, indicating the La Niña event that brought the lowest maximum and minimum summer air temperatures and third-highest summer precipitation quantity of the period 2000 to 2017^70^. Additionally and independently, Veettil et al.^70^ demonstrated that SLA at a Central Andes site rose rapidly in 2003, similar to the trend in our Fig. 2D, and when the ENSO was in a warm and dry phase and when simultaneously the Pacific Decadal Oscillation (PDO) was in its most positive phase of the period 2000 to 2015.

Rising glacier SLAs across the Central Chilean Andes is well-documented^71^ as is a rise in minimum glacier elevation^72^ summer snow cover decline^73^ and glacier mass loss^74^. Saavedra et al.^71^ reported SLA rise at 10 to 30 m.yr^−1^ south of 29–30˚S and those rates approximately correspond to 75th and 99th percentiles of our data for this region (10 and 40 m.yr^−1^, respectively). The higher rate of mean SLA_EOS_ rise in the Central Chilean Andes compared to the Central Andes has been attributed to reduced precipitation^71^ and indeed a ‘mega-drought’^75^ has persisted across the Central Chilean Andes since 2010, causing accelerated glacier thinning^76^ declined snow persistence^77^ and early-summer snowline rise^78^. Accordingly, the rate of change in SLA_EOS_ more or less doubled during 2011 to 2023 compared to the rate during 2000 to 2010, slightly more so in the east than in the west (Fig. 2E).

Our SLA_EOS_ anomalies were greatest in the Central Chilean Andes in 2009, 2015, 2018, 2021 and 2023, and lowest in 2003 (Fig. 2E), and concurrent with minimum summer snow extent in 2009 and 2015 for the 34–40˚S sub-region^73^. The timing of low SLA_EOS_ and maximum snow extent of 2003 corresponds to an El Niño period^73^ which, unlike in the Central regions at lower latitude, is associated with anomalously warm temperatures and high snowfall south of 29˚S^78,79^. The Central Chilean SLA_EOS_ highs of 2009 and 2015 (Fig. 2E) correspond with strongly positive Southern Annular Mode (SAM)^73^ and therefore weaker mid-latitude westerlies^80^ whilst minimum SLA_EOS_ in 2003 (Fig. 2E) correspond to strongly negative SAM, and stronger westerlies. In contrast, inter-annual variability in Central Chilean SLA_EOS_ compares less well to variations in ENSO phase between 2000 and 2015^74^, implying increased SAM conditions in the Central Chilean region^71^ and persistently so since 2018.

The SLA_EOS_ trends (Fig. 2F) and patterns (Fig. 3A, B, C) across the Southern Andes are the most complicated of all the Southern Hemisphere regions, illustrating marked contrasts in sub-regional climate and climate change. Rates of change in SLA_EOS_ were slightly positive for the North Patagonian Icefield (NPI: 2.78 m.yr^−1^) and South Patagonian Icefield (SPI: 1.49 m.yr^−1^) and negative for Cordillera Darwin (CD: −3.05 m.yr^−1^). The reduced rate of SLA_EOS_ rise in the SPI compared to the NPI corresponds to reports of SPI glaciers displaying neutral or positive mass balance, and/or stable or advancing extents in recent years^81–83^. Increasingly stable SLAs in the higher-latitude CD (Fig. 3C) can be explained by increasing precipitation^84^ that has arisen from southward displacement and intensification of westerlies at ~ 60˚S following increased SAM index and formation of anomalous low pressure near Drake Passage^80,85,86^.

Much of our observed inter-annual variability in Southern Andes SLA_EOS_ is consistent with reported variability in glacier change and reported climatic variability. For example, the low mean SLA_EOS_ and strongly negative SLA_EOS_ anomaly in 2001 (Fig. 2F) coincides with a positive summer precipitation anomaly and negative summer temperature anomaly observed in the Aysén river basin at 45–46˚S^87^. Pérez et al.^87^ also document 2001 as the year of maximum summer snow extent in Patagonia between 2000 and 2016. Our greatly reduced SLA_EOS_ in 2010/2011 (Fig. 2F) coincides with a sudden decrease in frontal ablation rates of SPI glaciers, Uppsala and Jorge Montt, in 2010/2011^89^ and is Likely explained by positive summer precipitation anomalies between 2009 and 2011, and a coincident negative summer air temperature anomaly in 2010^88^. Overall, our Southern Andes results imply that the well-reported poleward displacement of SAM-driven westerlies^73^ is causing increased aridity in the Patagonian Icefields and increased snowfall in Tierra del Fuego.

### Summary and conclusions

In summary, our study is novel in showing that SLA_EOS_ on Southern Hemisphere glaciers have been rising over the last two decades, and with an accelerating rate of rise in almost all mountain regions. Our quantification of temporal trends suggests large-scale region-wide warming but also influence of westerlies bringing precipitation to the west Southern Alps, west Antarctic Peninsula and western Andes. Inter-regional comparisons reveal teleconnections between Andean and Southern Alps glaciers; in-phase for northern Andes and out-of-phase for Patagonia^88^. Inter-annual variability (highly positive or negative anomalies) in regions such as the Antarctic Peninsula and Southern Andes suggests spatio-temporally complex climate systems, or confounding influences of warming and precipitation regime (solid v liquid) shifts. Elsewhere, such as in the Central Chilean Andes, consistently positive anomalies suggest that some climate system modes appear to be persistent and perhaps becoming stronger.

Given projected air warming for all regions^89,90^and projections of a strengthening SAM^91,92^ and thus intensified westerlies^93^ high- and mid-latitudes can expect summer drying^75,94–96^ and any increases in precipitation at lower latitudes will not alleviate glacier mass loss^97,98^. The frequency and intensity of ENSO phase transitions is also expected to amplify over coming decades^43^ which will affect the variability of drought conditions^79,99^ and blocking episodes^100^.

The most vulnerable glacier sub-regions to ongoing change, as indicated by our SLA_EOS_ observations, are (i) the eastern parts of the high-latitude ranges owing to downslope wind (e.g., foehn) driven by westerly wind intensification^101,102^ (ii) the Central Chilean Andes where both SAM and La Niña effects bring increased drought, and (iii) the maritime Southern Alps where marine heatwaves are set to intensify^75^ (iv) large parts of ice caps and icefields becoming exposed to ablation as SLA^EOS^ rises above plateau edges.

In conclusion, the upward and accelerating rate of rise of SLAs across the Southern Hemisphere that this study identifies and quantifies is likely to continue. Those rising SLAs and hence ELAs are forcing glaciers to be out of equilibrium with present day climate and to amount that is determined by glacier response times and with committed loss of ice. Unravelling the spatio-temporal patterns in SLA_EOS_ and hence ELAs will aid understanding glacier response times, committed ice losses and overall meltwater production in the coming decades, respectively.

## Supplementary Information

Below is the link to the electronic supplementary material.

Supplementary Material 1

## Data Availability

Glacier outlines are available from Global land Ice Measurements from Space via the National Snow and Ice Data Center (GLIMS and NSIDC, 2005, updated 2018) [http://glims.colorado.edu/glacierdata/](http:/glims.colorado.edu/glacierdata)Landsat Surface Reflectance at 30 m resolution from the U.S. Geological Survey is available from several data portals [https://www.usgs.gov/landsat-missions/landsat-data-access](https:/www.usgs.gov/landsat-missions/landsat-data-access)Elevation data at 30 m resolution is available from JAXA ALOS World 3D Digital Surface Model [https://www.eorc.jaxa.jp/ALOS/en/dataset/aw3d30/aw3d30_e.htm](https:/www.eorc.jaxa.jp/ALOS/en/dataset/aw3d30/aw3d30_e.htm)ERA-5 Land monthly aggregated data [https://confluence.ecmwf.int/display/CKB/ERA5-Land%3 A+data+documentation](https:/confluence.ecmwf.int/display/CKB/ERA5-Land%3 A+data+documentation)Drainage Basins are available from World Wildlife Fund HydrSHEDS [https://www.hydrosheds.org/](https:/www.hydrosheds.org).

## Code availability

An annotated copy of our SLA_EOS_ code for GEE is available at: https://code.earthengine.google.com/c578633c5a8b1d480eef09c97ec8fcb0.
