# Supplementary material for: Rising snowline altitudes across Southern Hemisphere glaciers from 2000 to 2023
Source: Sci Rep. 2025 Oct 13;15:35683. doi: 10.1038/s41598-025-19486-6 (PMC12518569; doi:10.1038/s41598-025-19486-6)
Supplement: Supplementary file 1 — Supplementary Material 1 [file 41598_2025_19486_MOESM1_ESM.docx]

**Rising snowline altitudes across Southern Hemisphere glaciers**

**from 2000 to 2023**

**Supplementary Information**

**Contents of this file:**

- Additional text on our datasets, methodological limitations, coverage of results, validation versus aerial surveys, comparison of SLA_EOS_ versus ERA5-Land climate trends
- Tables SI_1 to SI_4
- Figures SI_1 to SI_13

**Datasets**

Our datasets are listed in Table SI_1 and described below

Glacier outlines were obtained from GLIMS (RGI v.6) and Google Earth Engine (GEE) was used to retrieve the most recent outline feature for a given glacier. To reduce the processing runtime, glaciers with area between 1 and 100 km^2^ were selected for the Andes and Antarctic Peninsula, while glaciers 0.1-100 km^2^ were selected for the Southern Alps, which retained c. 6364 glaciers in total (Table SI_2). These thresholds were selected due to (i) higher relative frequency of smaller glaciers in the Southern Alps, and (ii) the comparative New Zealand aerial survey studying multiple index glaciers < 1 km^2^.

Atmospherically-corrected surface reflectance products from Landsat 4 TM, Landsat 5 TM, Landsat 7 ETM+, and Landsat 8 OLI were retrieved from the USGS via GEE. Landsat provides optimal combination of wide temporal coverage (images from 1983), high spatial resolution (30 m), and reasonable image acquisition intervals (16 days), hence its use in most automated snowline analyses (Li et al., 2022; Liu et al., 2021; Racoviteanu et al., 2019; Rastner et al., 2019). Previous work has suggested that top-of-atmosphere products may be preferential for use over the Antarctic Peninsula because of the uncertainty associated with deriving robust surface reflectance for polar regions (Chen and Zhu, 2022), but for consistency in this study surface reflectance was used throughout in line with previous automated snowline analyses (Li et al., 2022; Wang et al., 2023). Tier 1 products, with enhanced positional quality (Young et al., 2017), were obtained where possible but do not exist for the Antarctic Peninsula.

A digital elevation model (DEM) was required to build glacier contour grids and extract altitude measurements. ALOS World 3D 30 m (AW3D30; Tadono et al., 2016) digital elevation model (DEM) version 4.1 contains an elevation band with 30 m horizontal resolution. This DEM, which is a mosaic dataset composed of data from 2006 to 2011 and with a median date of 2009, was static (unchanged) throughout our study and workflow and was imported into GEE, clipped to our study glaciers and mosaicked to form a single-band raster. AW3D30 (version 3.2) has previously been used in automated snowline detection (Loibl et al., 2022), and is favoured over NASADEM (used in Li et al. (2022)) and Shuttle Radar Topography Mission (used in Racoviteanu et al. (2019) and Rastner et al. (2019)), due to its higher vertical accuracy (~5 m; Takaku et al. (2014)) and lower root mean square error (RMSE) (Bettiol et al., 2021; Mukul et al., 2017; Uuemaa et al., 2020).

Investigations into relationships of SLA_EOS_ with temperature and snowfall trends across the regions required meteorological data provided by ERA5-Land monthly aggregates (Muñoz-Sabater, 2019). Although this reanalysis dataset incorporates modelled data with observations and has relatively coarse spatial resolution (~11 km), its global coverage allows for consistency across study regions, which is less easily achieved with ground-based meteorological data from multiple sparsely located sources. In GEE, monthly snowfall and mean 2 m air temperature were averaged for (a) the Southern Hemisphere summer of a given year (December-March), and (b) the area over a given glacier. Gradient of change in climatic averages were calculated, for comparison to regional SLA_EOS_ trends.

Region-wide spatial variations in climate exist across each region, commonly exhibited in contrasting climates east and west of main (topographic) divides (Davies et al., 2012; Garreaud, 2009; Ummenhofer and England, 2007). Association of this climatic variation with glaciological variation was investigated by comparing the SLA_EOS_ trends for east-draining and west-draining glaciers. The drainage basin dataset from the Level 5 WWF HydroSHEDS dataset (Lehner and Grill, 2013) was used in GEE for the Southern Alps and Andes, and from the NASA Goddard Ice Altimetry Group dataset (Zwally et al., 2012) for the Antarctic Peninsula (Fig. SI_1).

| **Dataset** | **Product name** | **Data type** | **Spatial resolution (m)** | **Temporal resolution (days)** | **Reference** |
| --- | --- | --- | --- | --- | --- |
| Glacier outlines | Global Land Ice Measurements from Space (GLIMS) | Vector | 30 * | - | GLIMS and NSIDC (2005, updated 2013) |
| Annual end of summer snowlines  (this study) | U.S. Geological Survey (USGS) Landsat surface reflectance | Raster | 30 | 16 | - |
| Elevation | JAXA ALOS World 3D Digital Surface Model (DSM) | Raster | 30 | - | Tadono et al. (2016) |
| Monthly air temperature and summer snowfall | ERA5-Land Monthly Aggregated | Raster | 11,132 | 30 ** | Muñoz-Sabater (2019) |
| Major river drainage basins | World Wildlife Fund (WWF) HydroSHEDS basins | Vector | 500 *** | - | Lehner and Grill (2013) |
| Major river drainage basins | NASA Goddard Ice Altimetry Group | Vector | 500 *** | - | Zwally et al. (2012) |

*Assuming outlines digitised from 30 m satellite imagery. May not be applicable to all outlines.

**Approximation of monthly temporal resolution.

***Basins digitised from ~500 m raster data.

***Table SI_1 Datasets used in this study***

**
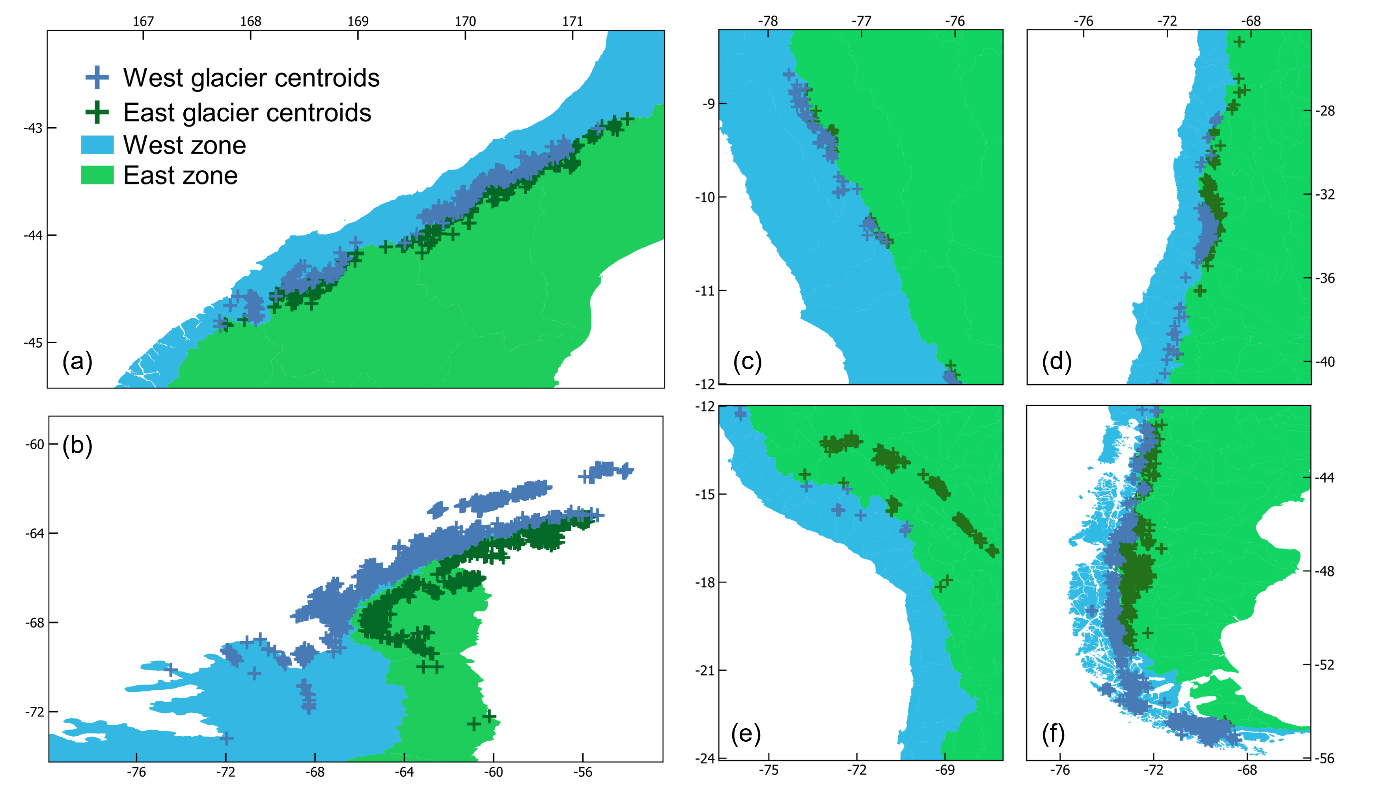
*Figure SI_1. Demarcation of our ‘east’ and ‘west’ sub-regions, as derived from drainage basins, in (a) Southern Alps; (b) Antarctic Peninsula; (c) Tropical Andes; (d) Central Andes; (e) Central Chilean Andes; (f) Southern Andes. The maps in this figure were made using ArcGIS Pro software*** [***https://www.esri.com/en-us/arcgis/products/arcgis-pro/overview***](https://www.esri.com/en-us/arcgis/products/arcgis-pro/overview)


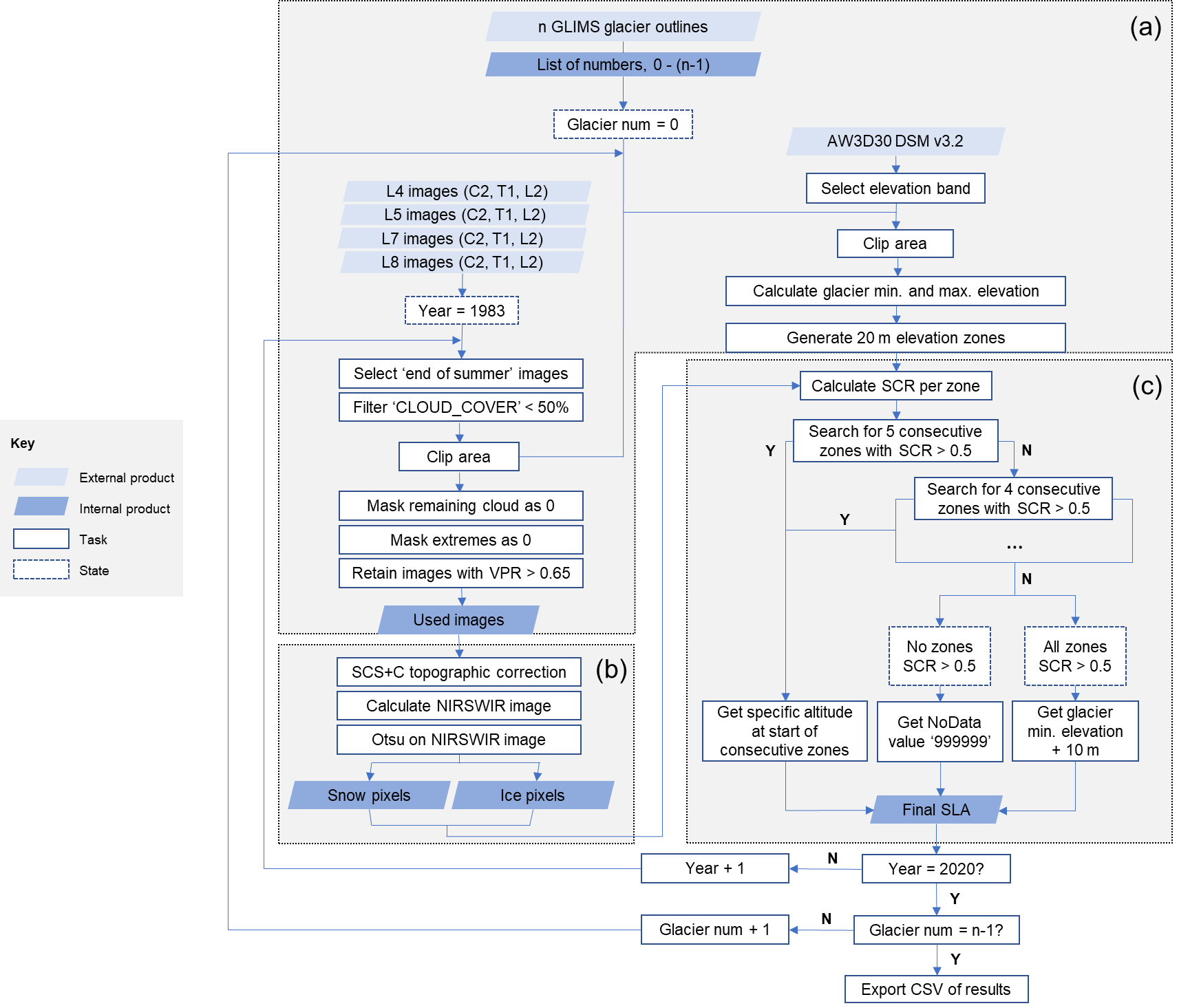


***Figure SI_2. Our processing scheme for SLA_EOS_ retrieval in GEE, which has been adapted from Li et al. (2022) to analyse multiple glaciers simultaneously. Part (a) is image and elevation data processing, (b) snow classification, and (c) SLA_EOS_ identification.***


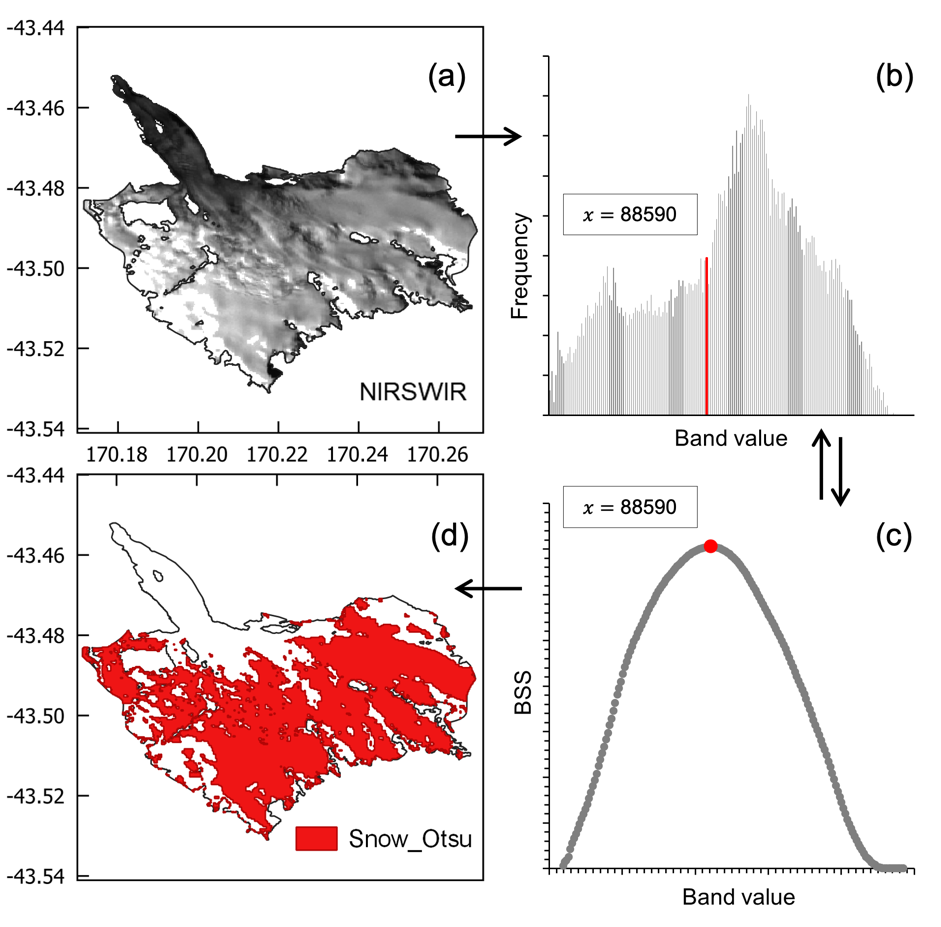


***Figure SI_3. Framework of Otsu algorithm application to identify case-specific snow classification threshold. (a) NIRSWIR band image of Franz Josef Glacier; (b) histogram of NIRSWIR image showing threshold (***$\boldsymbol{x}$***; red); (c) NIRSWIR value with maximum BSS (***$\boldsymbol{x}$***; red) corresponds to threshold in (b); (d) NIRSWIR pixels exceeding threshold value classified as ‘snow’. The maps in this figure were made using ArcGIS Pro software*** [***https://www.esri.com/en-us/arcgis/products/arcgis-pro/overview***](https://www.esri.com/en-us/arcgis/products/arcgis-pro/overview)

***
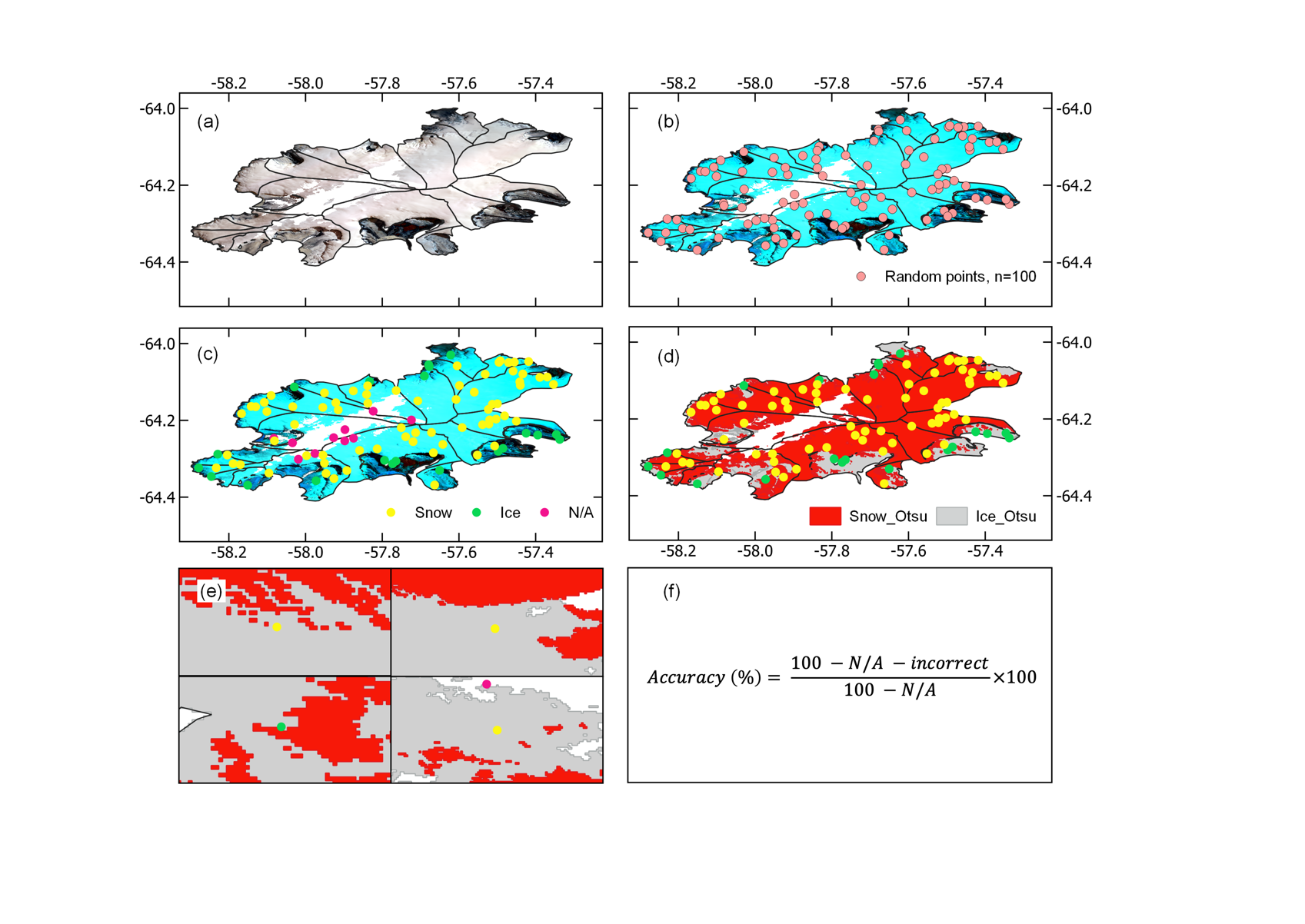
Figure SI_4. Framework of snow classification accuracy assessments using example from a region of James Ross Island, Antarctic Peninsula; (a) true-colour image from end-of-summer period of given year; (b) image converted to false-colour and 100 random sampling points overlaid; (c) using false-colour as guide, points designated into researcher’s interpretation of snow, ice, and N/A groups; (d) Otsu ‘snow’ and ‘ice’ classifications overlaid; (e) examples of incorrect Otsu classifications to be counted; (f) calculation of percentage accuracy using counts of N/A and incorrectly classified points. The maps in this figure were made using ArcGIS Pro software*** [***https://www.esri.com/en-us/arcgis/products/arcgis-pro/overview***](https://www.esri.com/en-us/arcgis/products/arcgis-pro/overview)

***
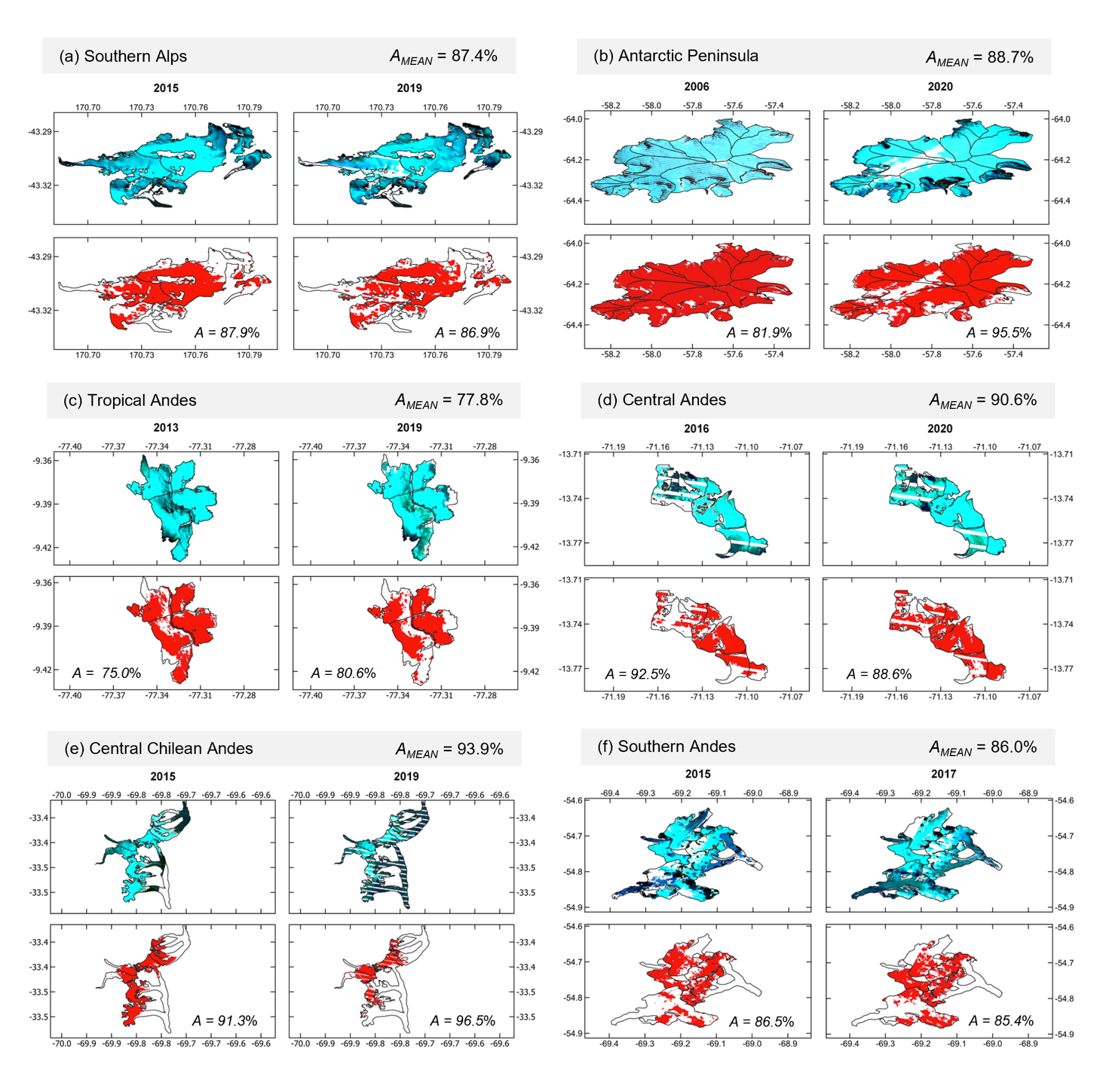
Figure SI_5. Inter-regional variation in mean accuracy (A_MEAN_) of automated snow classification (red), displayed with false-colour composites of images segmented by Otsu algorithm. Images taken from end-of-summer dates of the given year. Accuracy assessment sites were within: (a) Garden of Allah, Southern Alps; (b) James Ross Island, Antarctic Peninsula; (c) Cordillera Blanca, Tropical Andes; (d) Cordillera Vilcanota, Central Andes; (e) Cordillera Principal/Frontal, Central Chilean Andes; (f) Cordillera Darwin, Southern Andes. The maps in this figure were made using ArcGIS Pro software*** [***https://www.esri.com/en-us/arcgis/products/arcgis-pro/overview***](https://www.esri.com/en-us/arcgis/products/arcgis-pro/overview)

**Methodological limitations**

The 16-day repeat cycle of Landsat limits that the SLA_EOS_ is retrieved exactly on the date of it’s maximum altitude. In this study, multiple Landsat sensors with overlapping coverage (e.g., 5 TM and 7 ETM+ overlap from 2000–2011; 7 ETM+ and 8 OLI overlap from 2013–2020) were used to increase temporal resolution.

Use of 20 m elevation bands in SLA_EOS_ delineation also minimises the effect of not attaining maximum SLA_EOS_, as the maximum snowline can vary up to 20 m in the period that lacks imagery before a new elevation bin is designated as SLA_EOS_.

Due to cloud masking and subsequent removal of images with VPR < 0.65, SLA_EOS_ results for regions with higher cloud cover probability (Southern Andes, Tropical Andes and Antarctic Peninsula; Fig. SI_6) are largely sparse. Sparse results lead to regional SLA_EOS_ trends which may not be representative, as some annual SLA_EOS_ means were calculated from particularly small sample sizes of results. A minimum sample requirement of 10 glaciers was set for any calculation of regional annual mean SLA_EOS_, standard error (S.E.) bars were calculated to judge validity of SLA_EOS_ means, and clear anomalies in SLA_EOS_ mean were excluded from overall trends.

The CFMask algorithm used in cloud detection has inevitable difficulties over brighter surfaces, such as snow, and a tendency to overlook presence of optically thin clouds (Fog et al., 2017). Consequently, there was potential for undetected cloud cover to exist over glacier images, which may have been classified as ice due to low reflectance of cloud in the NIRSWIR band (Li et al., 2022). If cloud was present in an ablation zone, the effect on SLA_EOS_ calculation would be negligible; however, in a minority of cases, where cloud was present in the accumulation zone, snow cover would be underestimated and SLA_EOS_ overestimated. At glacier level examination, cloud presence could anyway be validated by its high reflectance in the SWIR band.

Finally, the presence of supraglacial debris and shadowing may have also limited the research strategy in some regions, as the typically darker areas would be classified as ice by Otsu. Where debris or shadowing exists in the ablation zone, its classification as ice will have made negligible difference to the snow classification and SLA calculation (Rastner et al., 2019). Darker surfaces existing in the accumulation zone will have instead caused underestimation of snow-covered area. Temporary ash debris cover visible in the snowline region of some Southern Alps glaciers, following the bushfire in the 2020 summer, caused snow cover underestimation and SLA_EOS_ overestimation (Fig. SI_8), whilst shadowing arising from steep and complex accumulation area terrain caused snow cover underestimation but did not notably affect SLA_EOS_ when shadowing was outside of the snowline zone (Figure 11b). The influence of darker surfaces was assumed to be negligible at hemispheric scale; for instance, debris-covered glaciers only comprise ~4% of global glacier area (Shukla et al., 2022), and the automated approach minimises the impact of anomalous pixels or images from the regionally averaged trends that are presented herein.

***
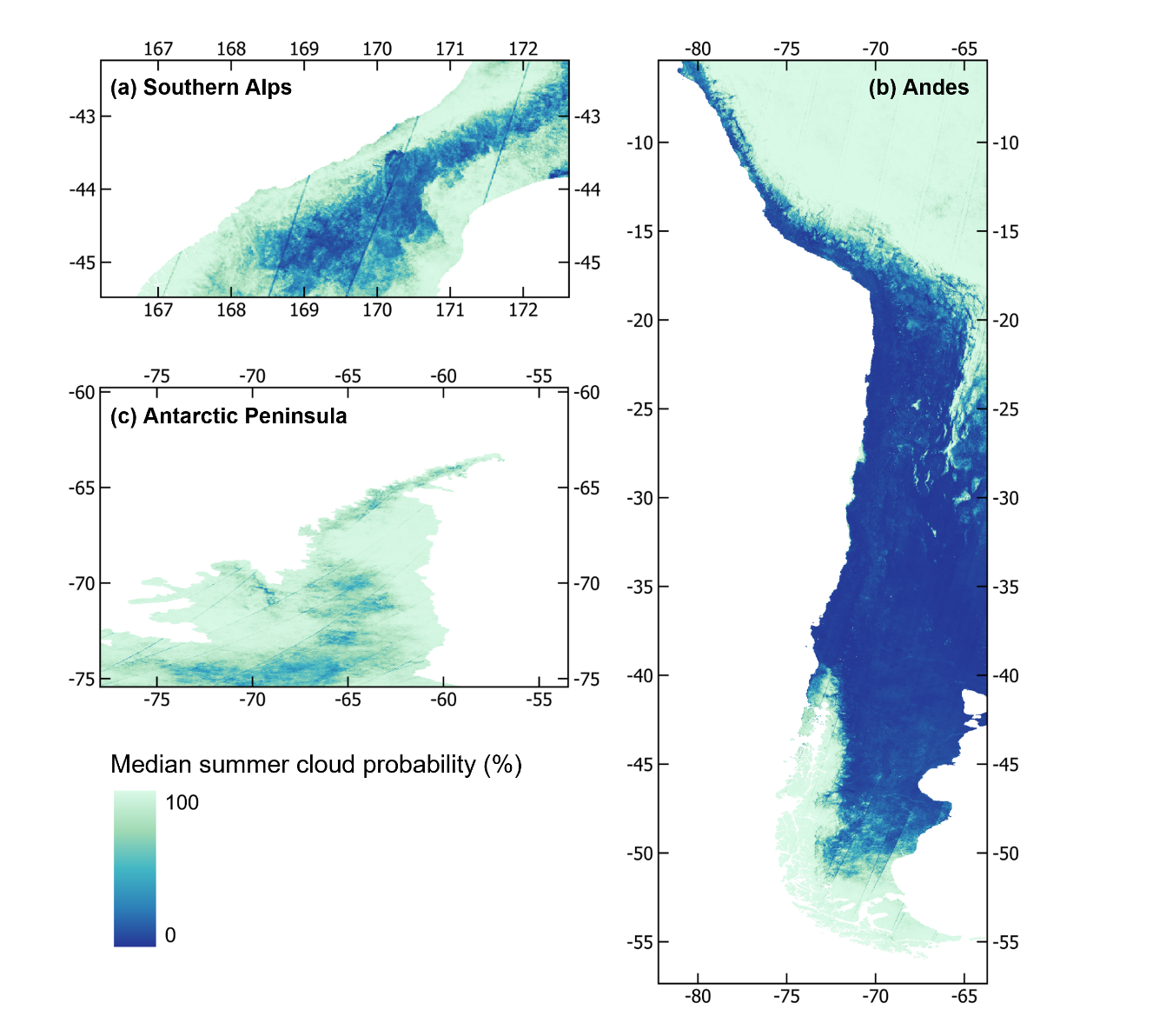
Figure SI_6. Median summer (December-March) cloud probability for the Southern Alps (a), Andes (b) and Antarctic Peninsula (c), derived from Sentinel-2 data (Copernicus Sentinel data, 2020). The maps in this figure were made using ArcGIS Pro software*** [***https://www.esri.com/en-us/arcgis/products/arcgis-pro/overview***](https://www.esri.com/en-us/arcgis/products/arcgis-pro/overview)


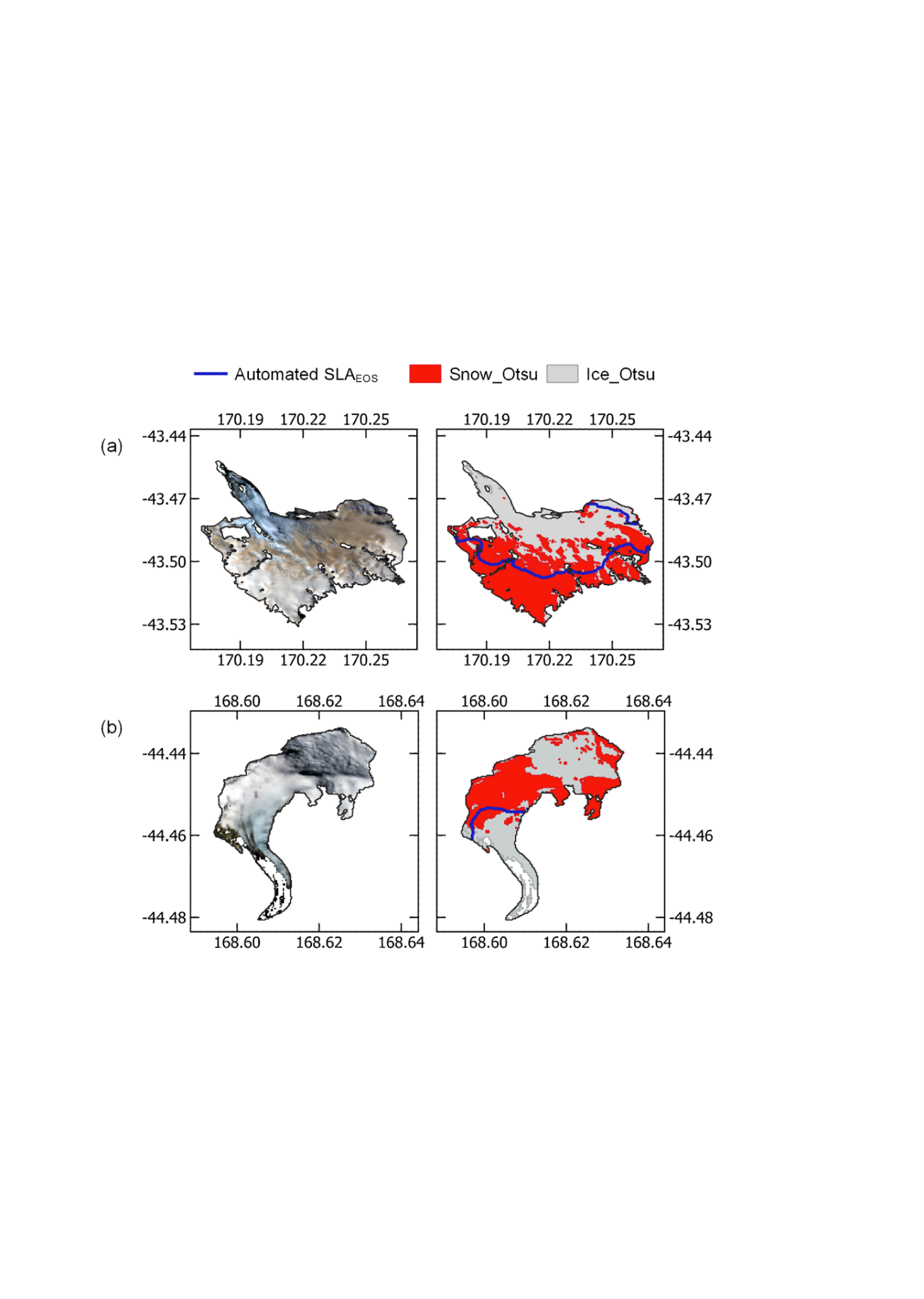


***Figure SI_7. Examples of glacier image scenes (left) which are problematic for automated snow classification and SLA_EOS_ delineation (right). (a) Temporary bushfire ash on Franz Josef Glacier, Southern Alps, causes SLA_EOS_ overestimation; (b) topographic shading on upper Dart Glacier, Southern Alps, causes snow/ice misclassification. The maps in this figure were made using ArcGIS Pro software*** [***https://www.esri.com/en-us/arcgis/products/arcgis-pro/overview***](https://www.esri.com/en-us/arcgis/products/arcgis-pro/overview)

**Coverage of SLA_EOS_ results**

Of the 6459 glaciers processed, 95 returned no SLA_EOS_ results from 1983-2020, leaving 6364 glaciers with results reported in this study. Table S3 describes the regional samples of glaciers following removal of glaciers with no SLA_EOS_ results.

In each region, the number of glaciers for which SLA_EOS_ results were retrieved was inter-annually variable during the period of 1983 to 2023 (Fig. SI_8). Indeed, no SLA_EOS_ results were retrieved for the Southern Alps pre-2000 (Fig. SI_8a), and the density of results was very low pre-2000 for the Antarctic Peninsula (Fig. SI_8b) and Southern Andes (Fig. SI_8f). Therefore, for consistency and to enable inter-regional comparisons, only SLA_EOS_ results for the time period 2000 to 2023 are reported.

For the period of 2000 to 2020, the count of annual SLA_EOS_ results retrieved per glacier varied spatially across the study regions (Fig. SI_9). Glaciers of the Central Chilean Andes had the highest density of SLA_EOS_ results across the 21 years (mean counts > 19 for majority of tessellation cells; Fig. SI_9b), followed by the Southern Alps (mean counts > 16 for majority of tessellation cells; Fig. SI_9a). The Tropical Andes and Antarctic Peninsula had a lower density of results (mean counts of 3 to 10 for majority of tessellation cells; Figs. SI_9b and 9c), and the Southern Andes had markedly lower density of results south of 50˚S (mean counts 3-7) compared to north of 50˚S (mean counts 10 to 21). Similarly, the Central Andes had a greater density of results in the west compared to the east.

| **Region** | **n** |  | **n West** | **%area** | **n East** | **%area** |
| --- | --- | --- | --- | --- | --- | --- |
| Southern Alps | 628 |  | 311 | 50 | 317 | 52 |
| Antarctic Peninsula | 1627 |  | 1169 | 10 | 458 | 4 |
| Central Andes | 431 |  | 41 | 21 | 431 | 78 |
| Central Chilean Andes | 566 |  | 209 | 47 | 357 | 67 |
| Southern Andes | 3112 |  | 1719 | 61 | 1393 | 48 |

***Table SI_2. Glacier sample sizes after glaciers without SLA_EOS_ results have been removed. % glacier area is of the RGI v6 inventory for that sub-region (E/W).***


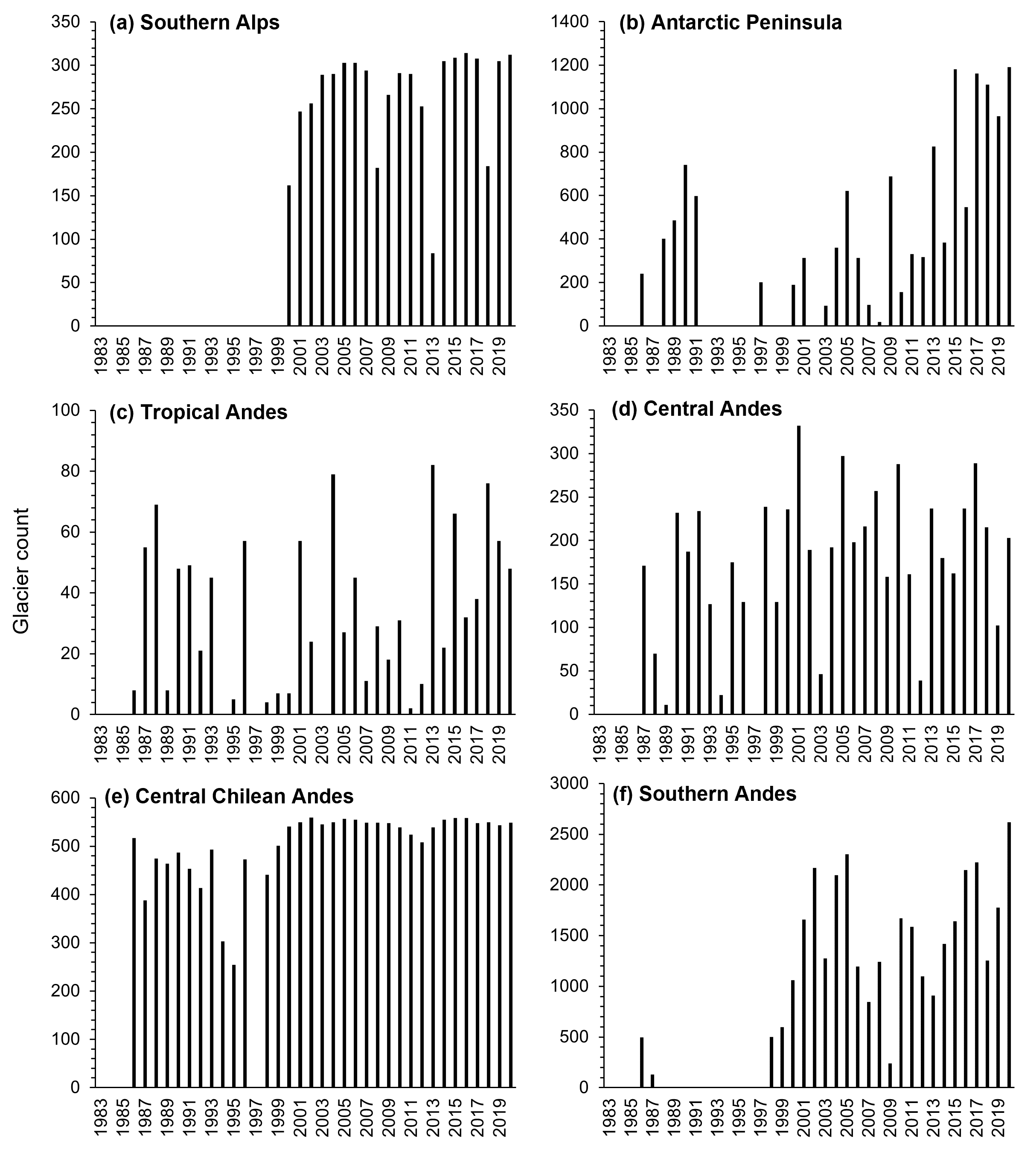


***Figure SI_8. Annual counts of glaciers with SLA_EOS_ results retrieved. Density of SLA_EOS_ results is lower pre-2000 for the Southern Alps, Antarctic Peninsula, and Southern Andes.***

***
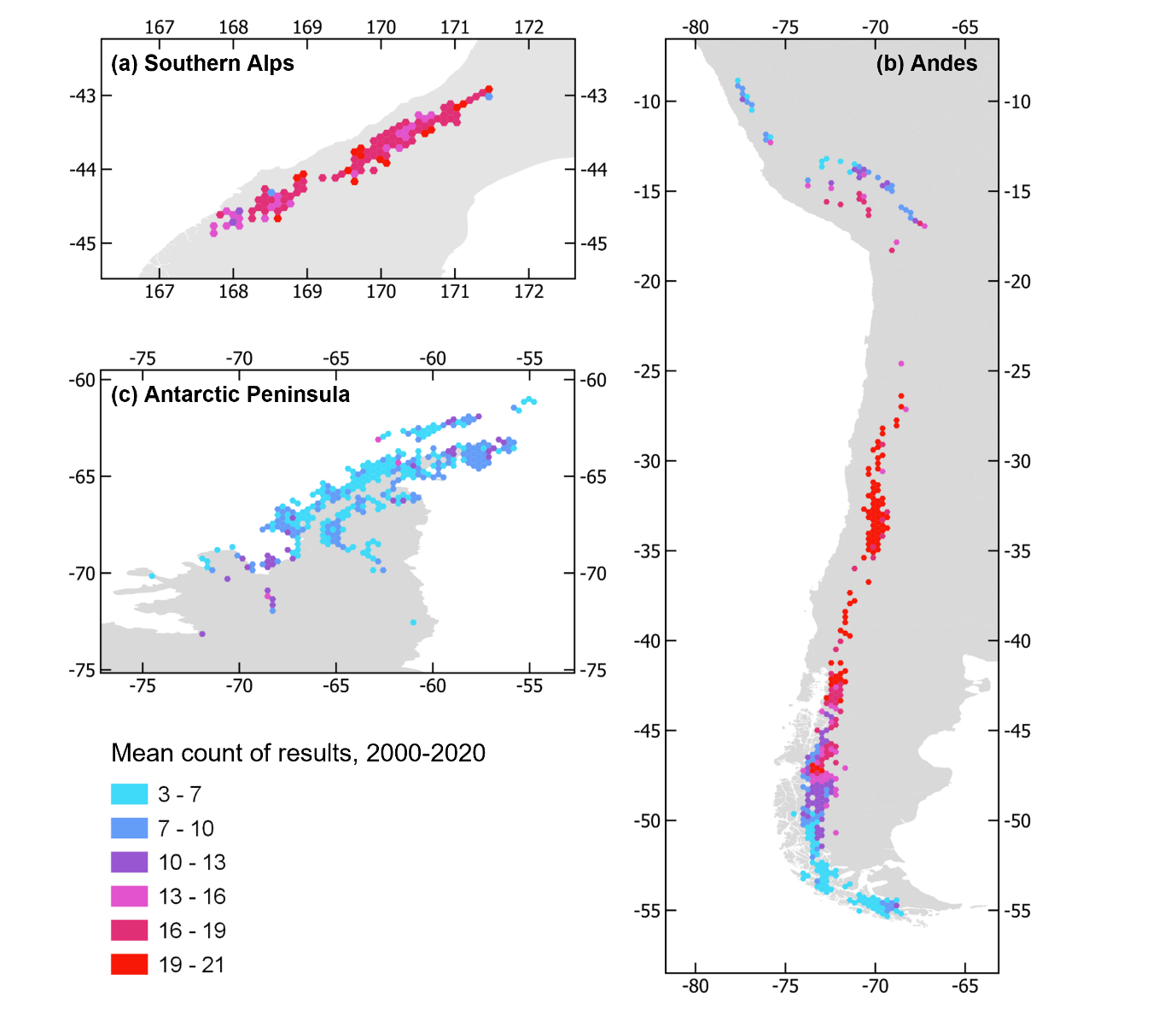
Figure SI_9. Spatial distribution of the SLA_EOS_ result density (count of years from 2000-2020 with results retrieved) across all regions, where each tessellation cell is the mean result count of glacier centroids within it. Highest quantity of results for the Central Chilean Andes and Southern Alps; lowest quantity of results for the Antarctic Peninsula, Tropical Andes, and Southern Andes. The maps in this figure were made using ArcGIS Pro software*** [***https://www.esri.com/en-us/arcgis/products/arcgis-pro/overview***](https://www.esri.com/en-us/arcgis/products/arcgis-pro/overview)

**Validation versus aerial surveys**

Based on an evaluation of the NDSI results and the NIRSWIR results versus the aerial survey records of SLAEOS (Fig. SI_10), using the NIRSWIR band ratio was rejected. Mean SLA_EOS_ for the Southern Alps test glaciers has a rising trend, which is in accordance with results produced by the New Zealand aerial survey (Fig. SI_11), and there is no statistically significant difference in SLA_EOS_ means determined by the two methods (Paired t-Test, p = 0.062). However, mean test glacier SLA_EOS_ was found to rise at a rate of 2.54 m yr^-1^ for the 21-year period, which is considerably lower than the 7.04 m yr^-1^ rate of change identified by the aerial survey method (Lorrey et al., 2022). Eastern test glaciers displayed a high rate of SLA_EOS_ rise (4.09 m yr^-1^) compared to the minimal SLA_EOS_ change detected for western test glaciers (0.81 m yr^-1^). This east-west contrast in rate also differs from the aerial survey findings, which indicated that eastern SLA_EOS_ increased at a lower rate (6.31 m yr^-1^) than western SLA_EOS_ (8.21 m yr^-1^) (Lorrey et al., 2022).


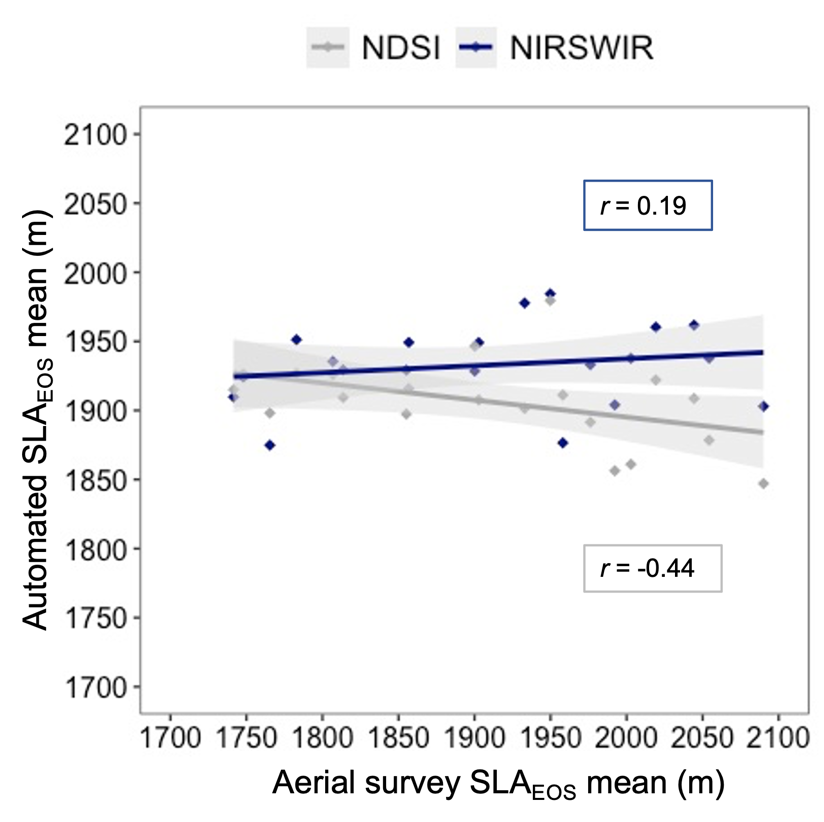


***Figure SI_10. Automated annual mean SLA_EOS_ of test glaciers (n=34) showed a non-significant moderate negative correlation (r=-0.44; p=0.053) to results collected by the New Zealand aerial survey (Lorrey et al., 2022) when NDSI image was used for snow classification, but non-significant weak positive correlation (r=0.19; p=0.43) to survey results when NIRSWIR image was used.***

***
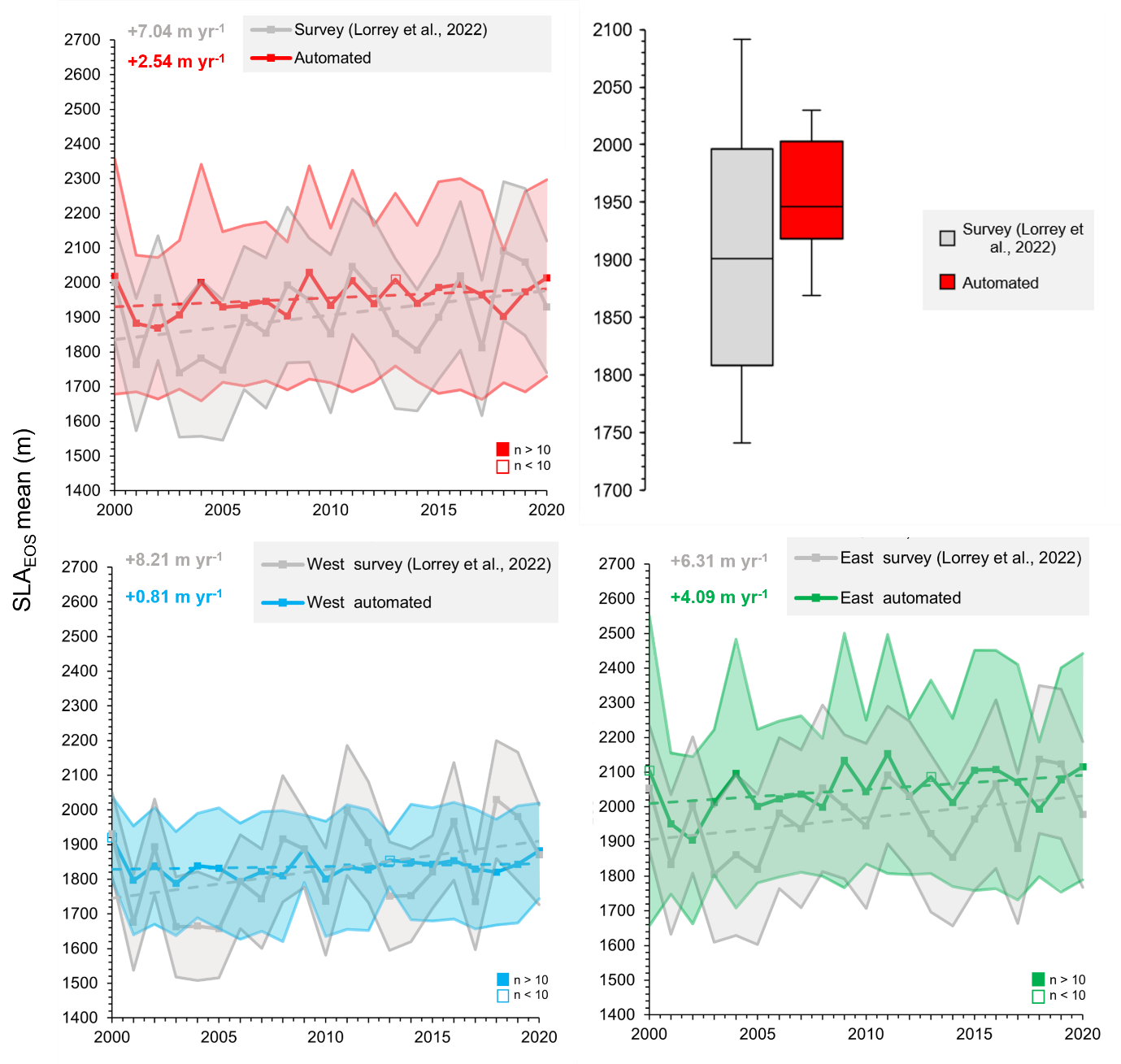
Figure SI_11. Comparison of automated SLA_EOS_ annual means with New Zealand aerial survey SLA_EOS_ annual means (Lorrey et al., 2022) for test glaciers (n =34). Top left: time series and trends of SLA_EOS_ change for all test glaciers. Top right: boxplot of variation in SLA_EOS_ means for all test glaciers. Bottom left: time series and trends of SLA_EOS_ change for west-draining test glaciers (n =15). Bottom right: time series and trends of SLA_EOS_ change for east-draining test glaciers (n =19). Hollow data points used when mean calculated from < 10 glaciers. Shaded areas represent ± 1 S.D.***

Despite disparities in rates of SLA_EOS_ change inferred by the two methods, the inter-annual variability in automated SLA_EOS_ remains within ± 1 standard deviation (S.D.) of aerial survey SLA_EOS_, with the exception of years 2004, 2005, and 2018 for western test glaciers (Fig. SI_11). Additionally, there were periods of inter-annual variability in mean SLA_EOS_ which accord with the pattern from aerial survey results: 2003 to 2005, 2009 to 2012, and 2014 to 2017.

Overall, automated SLA_EOS_ means were higher (median = 1947 m a.s.l.) than aerial survey SLA_EOS_ means (median = 1901 m a.s.l.) for the period 2000-2020 (Fig. SI_11). The range in aerial survey SLA_EOS_ was far greater than for automated SLA_EOS_; SLA_EOS_ maximum values were 2091 m a.s.l. (survey method) and 2030 m a.s.l. (automated method), while SLA_EOS_ minimum values were 1741 m a.s.l. (survey method) and 1869 m a.s.l. (automated method). The disparity in SLA_EOS_ variation in the two datasets is also shown in the higher mean S.D. of survey results (± 108 m) compared to automated results (± 48 m).

**Validation versus ELA from direct glacier mass balance measurements**

| Glacier | Sub-region | Measured ELA change (m)  1979 to 2018  (Ohmura and Boettcher, 2022) | Measured rate  (m yr^-1^) | This study local (tessellation cell as in Fig. 4a) SLA_EOS_ median rate of change  (m yr^-1^) 2000 to 2010 | This study local (tessellation cell as in Fig. 4a) SLA_EOS_ median rate of change  (m yr^-1^) 2011 to 2023 | This study sub-regional (Fig. 3) SLA_EOS_ median rate of change  (m yr^-1^)  2000 to 2023 |
| --- | --- | --- | --- | --- | --- | --- |
| Martial Este | S. Andes West | -96 | -2.4 | -1.5 | 20.6 | -1.0 |
| Chalcaltaya | C.Andes East | 204 | 4.7 | 12.8 | 6.5 | 6.1 |
| Zongo | Central Andes East | 120 | 3.0 | 15.4 | 18.3 | 6.1 |
|  |  |  |  |  |  |  |
| Hurd | AP West | -300 | -7.5 | 0 | 6.1 | -0.3 |
| Johnsons | AP West | -272 | -6.8 | 0 | 6.1 | -0.3 |
| Bahia del Diablo | AP East | -96 | -2.4 | -4.9 | -15.5 | 3.6 |

***Table SI_3. Comparison of changes in our SLA_EOS_ with changes in ELA calculated from measured/direct mass balance field data as reported in Ohmura and Boettcher (2022)***

***Figure SI_12. Comparison of change in our SLA_EOS_ with change in ELA from direct mass balance datasets, the latter from Ohmura and Boettcher (2022)***

**Comparison of SLA_EOS_ versus ERA5-Land climate trends**

ERA5-Land Monthly Aggregated climate data was retrieved and pre-processed in Google Earth Engine using the code: [https://code.earthengine.google.com/c3f6da4bc39753e0f06d8560d8e674b5](https://eur03.safelinks.protection.outlook.com/?url=https%3A%2F%2Fcode.earthengine.google.com%2Fc3f6da4bc39753e0f06d8560d8e674b5&data=05%7C02%7CJ.L.Carrivick%40leeds.ac.uk%7C5888eeb0aec9416975dd08dddc013b8d%7Cbdeaeda8c81d45ce863e5232a535b7cb%7C0%7C0%7C638908620070072635%7CUnknown%7CTWFpbGZsb3d8eyJFbXB0eU1hcGkiOnRydWUsIlYiOiIwLjAuMDAwMCIsIlAiOiJXaW4zMiIsIkFOIjoiTWFpbCIsIldUIjoyfQ%3D%3D%7C0%7C%7C%7C&sdata=Wxuw8GAwnnFuVwRUB%2BdwGBqRpuB5%2F%2BqnujZEbyNWpj4%3D&reserved=0) . Specifically, that code retrieves monthly air temperature and snowfall grids for the austral summer (Dec, Jan, Feb, Mar) and computes the cell by cell mean of them per year, for all mountain regions of the Southern Hemisphere.

That ERA-5 data indicated that mean 2 m air temperature was higher for the west Southern Alps, and monthly snowfall sum was higher for the east Southern Alps (Fig. SI_13a). Monthly snowfall sum lowered at -0.0002 m w.e. yr^-1^) in the west but increased by 0.0710 m w.e. yr^-1^ in the east, while warming occurred at a very similar rate in the east (0.0696 K yr^-1^) and the west (0.0710 K yr^-1^) (Table SI_4). Mean monthly summer snowfall was highest in 2003 and lowest in 2022, whilst mean summer temperature was lowest in 2004 and highest in 2022 (Fig. SI_13a).

The same climate data indicated that the western Antarctic Peninsula had higher overall summer air temperature, and higher summer monthly snowfall sum for most years (Fig. SI_13b). In both east and west sub-regions, temperature declined during 2000 to 2014 (-0.122 K yr^-1^ and -0.070 K yr^-1^ respectively), before increasing rapidly (0.244 K yr^-1^ and 0.309 K yr^-1^ respectively). Mean monthly summer snowfall decreased in the east (-0.0004 m w.e. yr^-1^) and increased slightly in the west (0.0008 m w.e. yr^-1^) sub-regions (Table SI_4).

ERA5-Land presented unambiguously higher summer snowfall in the eastern Central Andes (Fig. SI_13c), indicating that eastern snowfall was increasing (1.9488 m w.e. yr^-1^) whilst western snowfall was very slightly decreasing (-0.0002 m w.e. yr^-1^) (Table SI_4). Monthly summer snowfall was lowest in 2016 for both regions, and highest in 2000 for the west but 2011 for the east (Fig. SI_13c). Summer air temperature was higher in the western Central Andes, where warming also occurred at a greater rate (0.0189 K yr^-1^) than in the east (0.0116 K yr^-1^) (Table SI_4). Temperatures increased from minimum in 2000/2001 to maximum in 2016 (Fig. SI_13c).

The western Central Chilean Andes had higher overall summer temperatures and a greater rate of warming (0.0478 K yr^-1^) than the eastern sub-region (0.0327 K yr^-1^) (Fig. SI_13d; Table SI_4). In both sub-regions, temperatures were lowest in 2007 and highest in 2020. Climate data indicated the east to have higher monthly summer snowfall sums in 13 of the study years. Snowfall was highest in 2005 and lowest in 2020 (Fig. SI_13d), following very slight drying trends which occurred at a greater rate in the west (-0.0001 m w.e. yr^-1^) than the east (-0.00003 m w.e. yr^-1^) (Table SI_4).

Similar to the other Andean regions, ERA5-Land indicated mean summer air temperature to be higher in the west Southern Andes from 2000-2020 and mean monthly summer snowfall to be higher in the east in most years (Fig. SI_13e). Rates of warming were higher in the east (0.0441 K yr^-1^) than the west (0.0281 K yr^-1^) (Table SI_4); eastern temperatures varied from a minimum in 2001 to a maximum in 2008 but western temperatures were maximum in 2008 and minimum in 2014 (Fig. SI_13e). Summer snowfall decline occurred at a greater rate in the east (-0.0005 m w.e. yr^-1^) than the west (-0.0004 m w.e. yr^-1^) (Table SI_4); mean monthly summer snowfall was lowest in 2013 for both regions, but peaked in 2001 for the east and 2014 for the west (Fig. SI_13e).

***
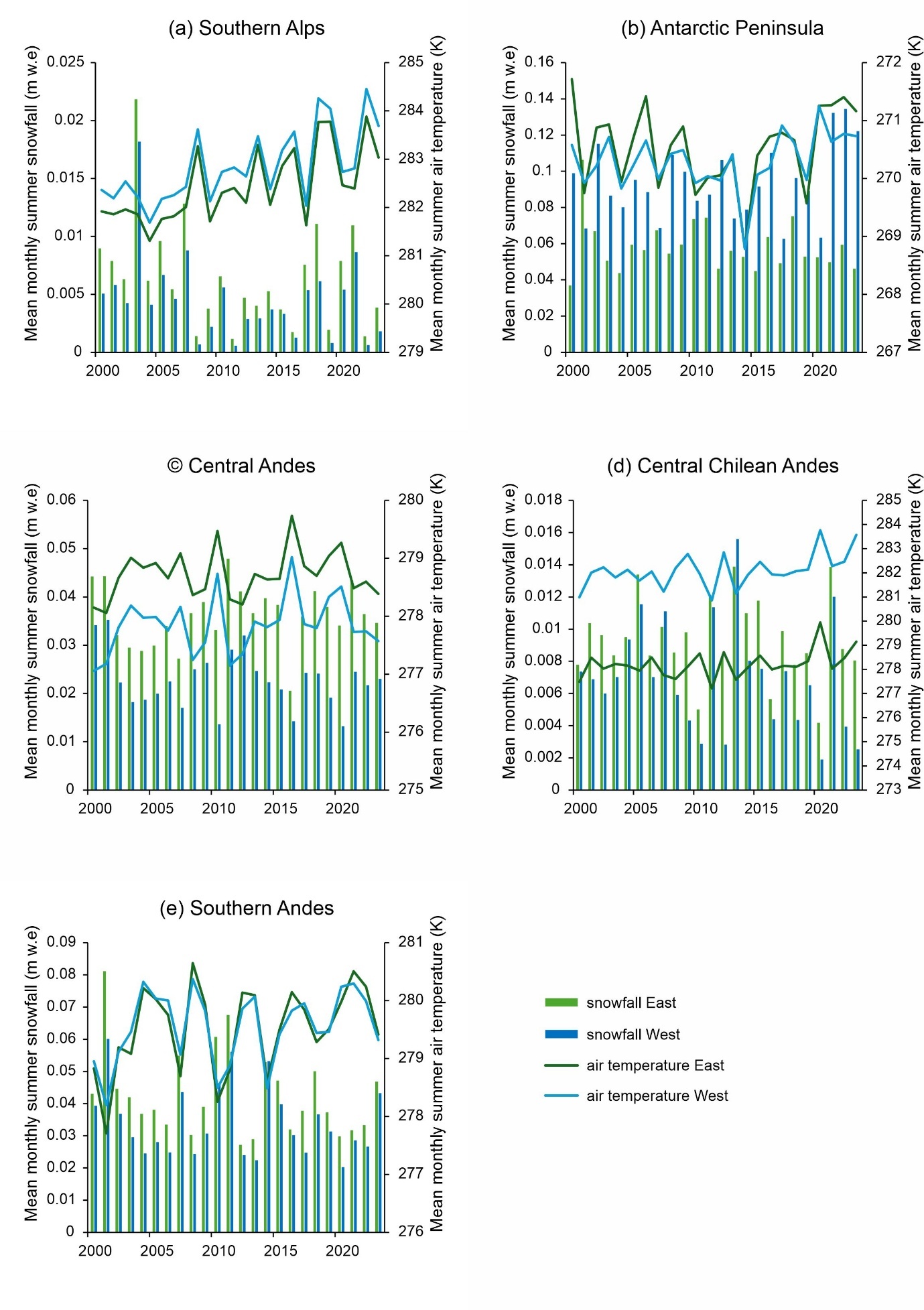
Figure SI_13. Mean summer (December-March) monthly snowfall sum and 2 m air temperature patterns for east and west sub-regions between 2000 to 2023.***

***Table SI_4. ERA5-Land Monthly Aggregated rates of change in mean monthly summer (December-March) 2 m air temperature and snowfall sum, for the east and west sub-regions from 2000 to 2023.***

| **Region** | **Rate of change in mean 2 m air temperature (K yr^-1^)** | | **Rate of change in mean monthly snowfall sum (m w.e. yr^-1^)** | |
| --- | --- | --- | --- | --- |
|  | **East** | **West** | **East** | **West** |
| Southern Alps | 0.0696 | 0.0710 | -0.0002 | -0.0002 |
| Antarctic Peninsula | 0.0086 | 0.0175 | -0.0004 | 0.0008 |
| Central Andes | 0.0116 | 0.0189 | 0.00008 | -0.0002 |
| Central Chilean Andes | 0.0327 | 0.0478 | -0.00003 | -0.0001 |
| Southern Andes | 0.0403 | 0.0260 | -0.0006 | -0.0003 |
